# Assessing Inequalities in Wellbeing at a Neighbourhood Scale in Low-Middle-Income-Country Secondary Cities and Their Implications for Long-Term Livability

**DOI:** 10.3389/fsoc.2021.729453

**Published:** 2021-11-08

**Authors:** Steve Cinderby, Diane Archer, Vishal K. Mehta, Chris Neale, Romanus Opiyo, Rachel M. Pateman, Cassilde Muhoza, Charrlotte Adelina, Heidi Tuhkanen

**Affiliations:** ^1^ Stockholm Environment Institute, Environment and Geography Department, University of York, York, United Kingdom; ^2^ Stockholm Environment Institute, Asia Centre, Bangkok, Thailand; ^3^ Stockholm Environment Institute, US Centre, Davis, CA, United States; ^4^ Department of Psychology, University Of Huddersfield, Huddersfield, United Kingdom; ^5^ Stockholm Environment Institute, Africa Centre, Nairobi, Kenya; ^6^ Stockholm Environment Institute, Tallinn Centre, Tallinn, Estonia; ^7^ Faculty of Biological and Environmental Sciences, University of Helsinki, Helsinki, Finland

**Keywords:** wellbeing, equity, urban, planning, livability, greenspace (Min5-Max 8), global south

## Abstract

To ensure future sustainability, cities need to consider concepts of livability and resident wellbeing alongside environmental, economic and infrastructure development equity. The current rapid urbanization experienced in many regions is leading to sustainability challenges, but also offers the opportunity to deliver infrastructure supporting the social aspects of cities and the services that underpin them alongside economic growth. Unfortunately, evidence of what is needed to deliver urban wellbeing is largely absent from the global south. This paper contributes to filling this knowledge gap through a novel interdisciplinary mixed methods study undertaken in two rapidly changing cities (one Thai and one Kenyan) using qualitative surveys, subjective wellbeing and stress measurements, and spatial analysis of urban infrastructure distribution. We find the absence of basic infrastructure (including waste removal, water availability and quality) unsurprisingly causes significant stress for city residents. However, once these services are in place, smaller variations (inequalities) in social (crime, tenure) and environmental (noise, air quality) conditions begin to play a greater role in determining differences in subjective wellbeing across a city. Our results indicate that spending time in urban greenspaces can mitigate the stressful impacts of city living even for residents of informal neighborhoods. Our data also highlights the importance of places that enable social interactions supporting wellbeing–whether green or built. These results demonstrate the need for diversity and equity in the provision of public realm spaces to ensure social and spatial justice. These findings strengthen the need to promote long term livability in LMIC urban planning alongside economic growth, environmental sustainability, and resilience.

## Introduction

With the global transition to urban living, cities need to become sustainable in the broadest sense, which increasingly includes concepts of wellbeing and quality of life alongside environmental and economic considerations (Leach et al., 2014). The Habitat III New Urban Agenda (NUA) includes a recognition that to maximize the benefits of urbanization we need to promote environmentally sustainable and resilient urban development (WHO, 2016; Habitat III Secretariat-United Nations, 2017). How to balance the need for urban environmental sustainability (which encompasses concepts of circular economies, resource conservation, and energy efficiency) which typically leads to densification, with the need for resilience (ability to withstand shocks and disasters), which entails diversity, remains an ongoing challenge (Elmqvist et al., 2019). The inclusion of considerations of wellbeing in urban sustainability entails that residents should not only live in a clean, safe and healthy spaces but should also have equity of opportunity to act and move around in health-promoting environments. In fast-changing cities, urban development can mean the loss of landcover supplying ecosystem services which provide multiple benefits in terms of the resilience to disasters, climate adaptation and support wellbeing (Derkzen et al., 2017). For future sustainability we need to better understand what ability different urban forms have for delivering these multi-functional benefits of promoting human-wellbeing, being environmentally sustainable and supporting resilience (Grimm et al., 2008; Hansen et al., 2017). This evidence is particularly lacking from the Global South where cultural and environmental conditions make their challenges and potential solutions distinct (Nagendra et al., 2018; Pauleit et al., 2021).

Rapidly developing cities in low-middle- income countries (LMIC) represent unique challenges and opportunities for the delivery of such sustainable development. The current rapid urbanization of sub-Saharan Africa is putting pressure on natural resources and the environment, increasing environmental- and climate change-related vulnerabilities, urban poverty and the proliferation of informal settlements (AFDB, 2013; UN-Habitat, 2015). These challenges are exacerbated by weak urban planning and management institutions, and inadequate urban governance (UN-Habitat, 2015; Smit, 2018). South East Asia is 49% urban while South Asia is at 36% (United Nations: Department of Economic and Social Affairs Population Division, 2019). However, these percentages are increasing faster than urban infrastructure provision leading to over 130 million South Asian living in informal settlements (Ellis and Roberts, 2016) and facing problems of inadequate housing, poor air quality and sanitation. Meanwhile, the Asia Pacific region is one of the most exposed to the changing climate, and is projected to see extremes in precipitation, temperature, and sea level rise, with the associated economic, social and physical costs (Asian Development Bank, 2017) including in urban contexts.

Unfortunately, such unplanned growth often outpaces infrastructure provision and occurs at the expense of a city’s ecological foundations, undermining resident’s wellbeing and the city’s sustainability (McPhearson et al., 2016). Addressing this entails moving beyond concentrating on only meeting basic needs towards enabling residents to achieve their aspirations. Developing more sustainable cities does not merely concern the improvement of infrastructure and systems mediating urban life, but also needs to consider the social aspects of city living, such as people’s satisfaction, experiences and perceptions of their everyday environments (Corburn, 2017; Shackleton et al., 2021). Achieving this requires that city authorities take sympathetic care of residents (Winkler, 2012) meaning decision makers need to have a greater understanding of a cross-section of their people’s needs and wants, aiming for ideals of equity, equality, social and spatial justice most relevant in LMICs (Soja, 2010; Bai et al., 2018; Zuniga-Teran and Gerlak, 2019).

Wellbeing supporting environments that promote mental health allow individuals realize their own abilities, cope with the normal stresses of life, work productively and fruitfully, and contribute to their community (World Health Organization (WHO), 2014). Research is increasingly demonstrating the importance of immersion in nature for health including both mental and spiritual wellbeing and physical health (both from direct opportunities afforded for recreation and socializing (Bertram and Rehdanz, 2015; Ahirrao and Khan, 2021) but also urban agriculture). Studies, including some from the Global South, indicate using urban greenspace can reduce residents stress (Roe et al., 2013; Adhikari et al., 2019), improving cognitive performance (Berman et al., 2008; Dadvand et al., 2015), decreasing depressive symptoms (Bratman et al., 2015) and increasing relaxation (Neale et al., 2019). Even if not directly accessing natural spaces, all urban residents can reap the benefits of regulating services provided by green and blue infrastructure such as shade cooling, air quality improvement, noise buffering or flood mitigation that again connect to physical and mental health. However, rapidly developing and changing urban environments, driven by desires to maximize land use, means that urban greenspaces are often converted into built and paved areas. The negative impacts of reducing urban nature are long-term; difficult to reverse; and increasingly important as cities develop. This link between human and ecosystem health is conceptualized as “Ecological Public Health” which represents the complex interactions between humans and the urban biosphere. A recent review concluded “better informed decisions using neighbourhood-level health determinants datasets stand to improve the environments and societies in which we live, particularly in LMICs” (Thomson et al., 2019) supporting calls from previous studies (Nero, 2017).

This paper explores these multiple dimensions of city developments impacts on resident’s wellbeing in LMIC contexts. We present results from two complementary LMIC cities exploring the interaction of urban form on wellbeing. Our findings address knowledge gaps that call for greater granularity of data to explore interactions with income, gender and environment (Patel et al., 2017). Our analysis considers the equity implications of this relationship contributing to recommendations for future city development pathways in LMIC settings to maximize sustainability that incorporates concepts of livability and wellbeing.

### Research Questions

We addressed these topics in relation to three interlinked questions:

1. How are objective aspects of wellbeing (distributed according to socioeconomic and sociodemographic characteristics) related to subjective assessments of wellbeing (life satisfaction)?
2. How is the relationship between subjective wellbeing mediated by the quality of urban environments?
3. What are the implications for urban development to achieve equitable wellbeing improvements?

## Methods

### Case Study Site Selection

**FIGURE 1:**
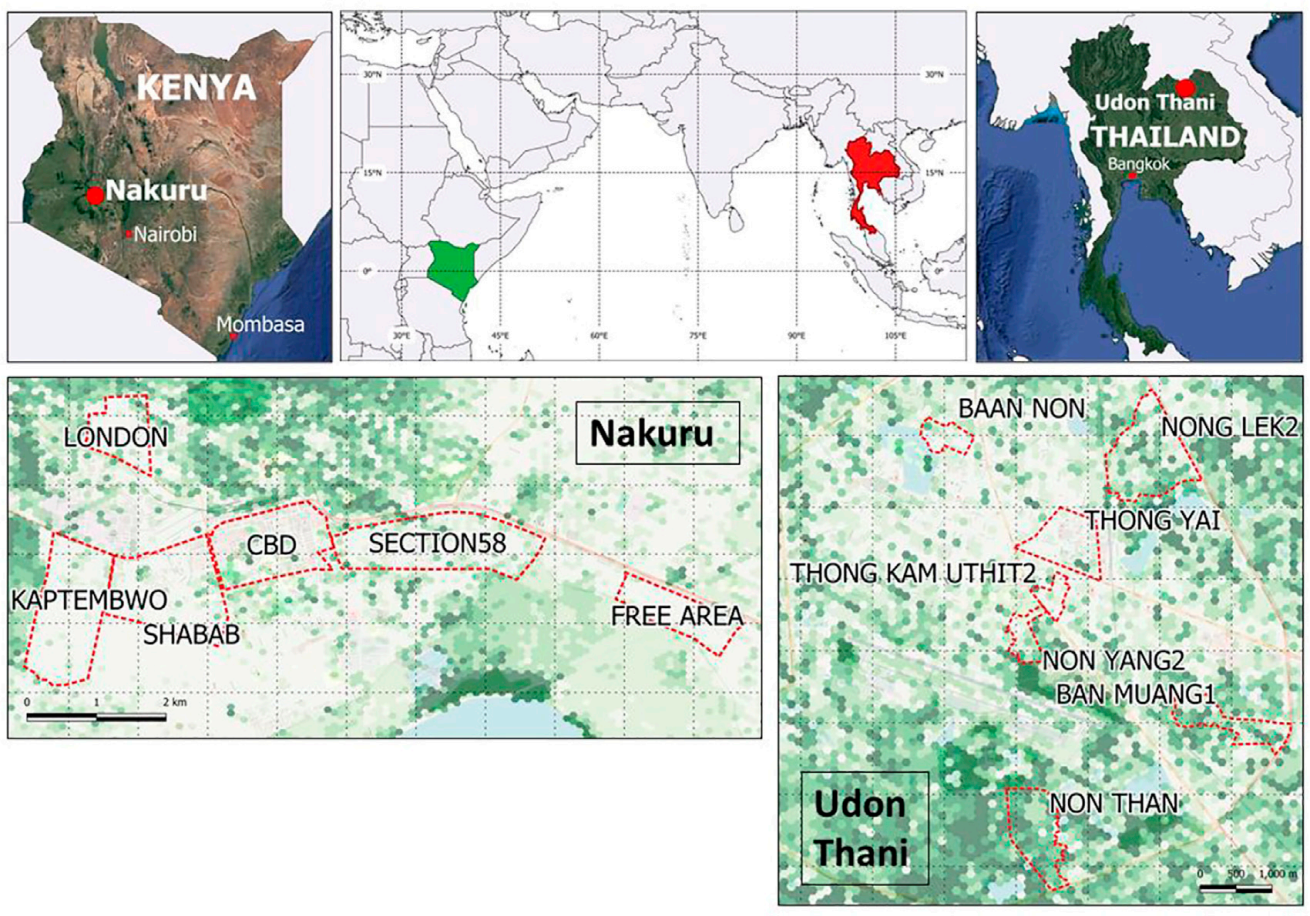
case study city locations and surveyed neighbourhoods. Base maps indicate 100 m width hexed grid relative greenness derived from Landsat imagery processed to show Normalised Difference Vegetation Index (NDVI).

To investigate these questions in real world settings two comparable but contrasting secondary cities of the Global South were selected (based on criteria including population and growth rates, mix of formal and informal growth, range of environmental concerns, relatively under researched) as representative examples in which to explore these concepts (see Figure 1 for details).

Nakuru, located within the Great Rift Valley, 160 km northwest of Nairobi, is the fourth-largest city in Kenya (after Nairobi, Mombasa and Kisumu) and the county capital. Nakuru lies at an altitude of 1,850 m and has a Mediterranean climate (Köppen-Geiger climate classification is Csb) remaining temperate throughout the year with no annual dry season. According to the County Integrated Development Plan 2018-2022, Nakuru town had an estimated population of 405,000 in 2018 which is expected to reach 458,000 by 2022 (a 13% increase). It has a mixture of built environments, including informal and unplanned settlements and both green and blue spaces. Rapid growth in Nakuru is putting development pressure on the public realm including greenspace.

Udon Thani in northeast Thailand is a small city of 130,000 residents facing rapid development due to its strategic location near the Laotian border. The city has a tropical savanna climate (Köppen-Geiger classification Aw) with warm dry winters followed by a 6-month monsoon season. Through the Udon Charter for 2029, a multi-stakeholder vision for the city, the city is committed to achieving six policy points, driven by the objective of becoming a green city focused on MICE (Meetings, Incentives, Conventions and Exhibitions). It seeks to have a walkable urban core, invest in green transport and green infrastructure including parks and public realm spaces.

### Surveys

Wellbeing can be considered a key component for a person’s quality of life and encapsulates both objective and subjective elements. The objective dimensions define wellbeing in terms of quality-of-life indicators including access to basic needs resources (e.g. food, housing, income) and social attributes (education, health, political voice, social networks). The subjective dimension emphasizes people’s own life evaluations including satisfaction (a cognitive evaluation) and happiness (relative emotional state) (Western and Tomaszewski, 2016). Subjective wellbeing encompasses hedonic functions such as pleasure attainment and pain avoidance, and eudemonic linked to a meaningful existence related to personal functioning (within individuals own mental and physical constraints) (Nordbakke and Schwanen, 2013).

This paper reports on the findings from two surveys detailed below: a bespoke neighbourhood survey investigating dimensions of socio-economic, environmental and wellbeing conditions (see 2.2.4.1 and 2.2.4.2 below); and a validated scale questionnaire exploring individual mood effects in different urban settings (see 2.2.4.3). All the survey tools received individual ethical approval via the relevant University of York, UK committee and participants gave informed consent. To facilitate accurate completion surveys were translated into local languages appropriate for each city.

#### Neighbourhood Wellbeing Survey Recruitment and Data Collection

The wellbeing survey was carried out across diverse neighbourhoods (six in Nakuru (during November 2018 dry season) and seven in Udon Thani (during December 2018 warm season)), identified in collaboration with city officials and local project partners, which represented a cross-section of local environmental, social and economic conditions ranging from central to suburban locations, including fully to partially serviced areas in terms of public utilities neighbourhood (see supplementary materials: two Assessment of socio-economic conditions). Adults (over the age of 18) were recruited through on-street intercepts in each neighbourhood aiming for a gender balanced sample.

##### Urban Settings Survey and Data Collection

To assess the impact of different types of urban spaces upon mood, a young (18–30 years) gender balanced, self-reported healthy, cohort of residents were recruited. This cohort was purposively selected to control for impacts of ageing on mobility and wellbeing as these participants also undertook recordings of heart rate variability (reported on in an upcoming paper) in different urban locations. Participants undertook transect walks between a busy built public realm space (market) and a quieter greenspace (public park) via other important infrastructure (e.g. bus interchange). These start and end points were selected to maximise the contrast in terms of type of public realm space–green vs grey; busy vs quiet. To control for the effects of direction the cohort was randomly sub-divided to undertake the walk in opposing directions (see supplementary materials part 2: Assessment of socio-economic conditions). Transect walks and mood surveys were undertaken in april 2019 during Udon Thani’s hot season and Nakuru’s wet season. Walks were only undertaken on dry days and in early morning to avoid high temperatures.

#### Assessments of Objective Wellbeing

The neighbourhood survey included questions on the impact on respondent’s wellbeing of eleven different environmental and social factors. The impacts ranged from large (scored 1) to no impact (4) on a forced four-point Likert scale. By summing the participant’s response scores across the eleven variables, a composite indicator of objective wellbeing was created. The raw data was scaled for graphing to range between greater than zero and the range maximum by subtracting the integer value of the minimum objective wellbeing score for each city. This improves visualization but means the graphed values are city specific and should not be directly compared (see supplementary materials:

To assess the relative affluence of the different surveyed neighborhoods the calculated mean sum of the ranked values for homeownership, employment status and job description were used. Job description was rated from employee upwards through managerial to business owner or professional. Two independent variables were used to validate the composite indicator of affluence, namely relative access to sanitation and access to water.

#### Assessments of Subjective Wellbeing

##### Wellbeing

The neighbourhood survey (translated into local languages as appropriate) utilized the Short Warwick Edinburgh Mental Wellbeing Scale (SWEMWBS) that assesses subjective wellbeing through seven questions rated on a five-point Likert scale which have been validated for construct validity (Stewart-Brown et al., 2009). The scale has revealed national wellbeing averages in the UK (McFall and Garrington, 2011) and successfully used in Europe (Koushede et al., 2019), Asia and Africa (Neale et al., 2019). This scale asks respondents to consider dimensions of life related to their wellbeing over the past 4 weeks.

##### Perceived Stress

Stress is inevitable and healthy factor of life. However, the duration and frequency of stress as well as someone’s belief and ability to return to a non-stressed state has significant implications for overall health and wellbeing. The Perceived Stress Scale (PSS), is a measure of sub-chronic stress (Cohen, 1983) which evaluates subjective levels of stress over the previous 2 weeks. Survey questions were designed to measure how unpredictable, uncontrollable, or overloaded respondents find their lives. The PSS has been used successfully in African and Asian contexts (Cohen, 1983; Neale et al., 2019) making it appropriate for this cross-cultural assessment. Higher scores on the PSS refer to higher stress (which is problematic) and on SWEMWBS to higher wellbeing (which is beneficial).

##### Mood

The urban settings survey used the Acute Subjective Mood measured by the University of Wales Institute of Science and Technology (UWIST) Mood Adjective Checklist (MACL) to determine acute subjective mood changes between our two key locations (market and park). MACL is a 24-item checklist that gives an acute psychometric measure of hedonic tone (valence), stress and (physical) arousal, shown as three scores. Respondents are required to complete the questionnaire before and soon after completion of activity to ensure measurement of momentary shifts in mood. The arousal scale measures feelings of subjective energy. The stress scale measures feelings of subjective tension and the hedonic tone scale measures overall pleasantness of mood and is associated with feelings of somatic comfort and wellbeing. Scores are obtained from summation of individual item scores pertaining to each of the three mood components.

The age and gender distribution of survey participants in each city can be seen below in table 1 (also see supplementary materials part 1: Detailed breakdown of participant numbers by neighbourhood).

**TABLE 1:** Survey participant demographics (Note: Thailand median age 40.1 yrs vs Udon Thani Neighbourhood wellbeing survey median age 46.26 years; Kenya median age 20.1 year vs Nakuru Neighbourhood wellbeing survey median age 41.96 years (country demographic information from worldometers.info Sep 2021). The mean age of the UWIST surveys was in Nakuru, 22.8 years for women, 24.6 years for men; Udon Thani, 24.1 year for women; 24.7 years for men)*.*

Survey		Participant demographics (W=Women/M = Men)							Survey description
		Neighbourhood	Age						
			18–30	31–45	46–60	61–75	76+	Total	
Neighbourhood Wellbeing Survey	Nakuru	CBD Total:57	M:8 W:8	M:13 W:10	M:7 W:4	M:5 W:1	M:1 W:0	M:34 W:23	Likert scale questions; Short Warwick Wellbeing; Perceived Stress; Use of green and public realm space
		Free Area Total:78	M:13 W:11	M:12 W:14	M:9 W:9	M:2 W:6	M:2 W:0	M:38 W:40	
		Kaptembwo Total:130	M:19 W:20	M:22 W:23	M:14 W:16	M:9 W:4	M:2 W:1	M:66 W:64	
		London Total: 97	M:16 W:19	M:15 W:17	M:8 W:14	M:9 W:10	M:4 W:1	M:52 W:45	
		Section No58 Total:100	M:15 W:13	M:16 W:13	M:14 W:12	M:7 W:5	M:3 W:3	M:55 W:45	
		Shabab Total:50	M:7 W:7	M:7 W:10	M:7 W:5	M:3 W:1	M:1 W:2	M:25 W:25	
		TOTAL:528	M:78 W:78	M:85 W:87	M:59 W:60	M:35 W:26	M:13 W:7	M:270 W:258	
	Udon Thani	Baan Non Total:64	M:7 W:10	M:9 W:9	M:6 W:12	M:8 W:6	M:0 W:0	M:27 W:37	
		Thong Yai Total:136	M:16 W:8	M:19 W:20	M:27 W:22	M:8 W:13	M:8 W:3	M:70 W:66	
		Baan Muang 1 Total:90	M:8 W:7	M:11 W:14	M:12 W:20	M:7 W:10	M:1 W:0	M:39 W:51	
		Thongkham Uthit 2 Total:91	M:9 W:8	M:19 W:16	M:13 W:9	M:7 W:7	M:1 W:2	M:49 W:42	
		Non Yang 2 Total:39	M:5 W:5	M:3 W:4	M:3 W:10	M:1 W:6	M:0 W:2	M:12 W:27	
		Nong Lek1&2 Total:80	M:8 W:10	M:7 W:12	M:10 W:26	M:5 W:1	M:0 W:1	M:30 W:50	
		Non Than Total:87	M:6 W:7	M:6 W:12	M:18 W:14	M:18 W:3	M:3 W:0	M:51 W36	
			TOTAL:587	M:59 W:55	M:74 W:87	M:89 W:113	M:51 W:46	M:5 W:8		M:278 W:309
Transect Walk		City	W	M	Total				UWIST Mood Adjective Checklist
		Nakuru	58	64	122				
		Udon Thani	58	57	115				

#### Assessments of Urban Infrastructure

Natural urban spaces, often referred to as urban greenspace (UGS), have been defined as vegetated urban spaces (Taylor and Hochuli, 2017). Whilst this definition is not globally appropriate as it prioritizes green–for our case study locations climatic-ecological settings it remains relevant for our analysis. To evaluate the impact that urban infrastructure availability and use has on wellbeing two data sources were utilised. Firstly, the participant’s response to questions on accessibility (do you live within walking distance of … ) and how much time they spend in these location (how many hours do you spend in these spaces (both within and beyond walking distance)) of greenspace and built public realm spaces. A walking ‘distance’ of 15 min was given as a guide to the participants in answering the accessibility question. Secondly, to quantify greenspace, satellite imagery pre-processed to indicate mean normalised difference vegetation index (NDVI) values for the year our survey was undertaken was obtained from Climate Engine which uses Google’s Earth Engine for on-demand processing of satellite data.

### Spatial Analysis

To assess the quantity of greenspace satellite imagery processed to depict vegetation (normalized difference vegetation index (NDVI)) was accessed. Landsat imagery was processed by Climate Engine (climateengine.org) to determine the mean NDVI values for the 12 months prior to the survey period to assess the most recent variations in greenness that could affect wellbeing. These images were clipped to official neighbourhood boundaries for both cities and the distributions of 29 m pixel values determined for input to statistical tests.

### Statistical Analysis

One-way ANOVA and Chi2 tests were utilized in IBM SPSS Version 26 to assess the differences between variables based upon age, gender and location. Tukey and Cramer V post-hoc tests determined the significance of any emerging associations or differences. Linear regression analysis was used to assess the explanatory strength of relationships between variables. Kruskal-Wallis H test was used to assess the differences in the distribution of NDVI pixel values by neighbourhood.

## Results

### Objective Wellbeing Dimensions

The following sections results present findings relevant to our initial research question of ‘how are objective aspects of wellbeing (distributed according to socioeconomic and sociodemographic characteristics) related to subjective assessments of wellbeing (life satisfaction)?’ All the significant statistical analysis presented in this results sections are in table 2 below.

**TABLE 2:** Statistical analyses underpinning the results.

Esults section	Variables compared	Statistical result	
3.1.2 Nakuru economic and socio-environmental conditions	Chi2 test of Employment status and Neighbourhood	χ2 (10) = 28.191, *p* = 0.002	
	One-way Anova comparison of Objective Wellbeing Score between Kaptembwo and Shabab	F (5, 506) = 3.282, *p* = 0.006	
	One-way Anova comparison of Women’s Objective Wellbeing Score by Neighbourhood	F (5, 242) = 3.396, *p* = 0.006	
3.1.3 Udon Thani economic and socio-environmental conditions	Chi2 test of Tenancy status and Neighbourhood	χ2 (6) = 26.810, *p* < 0.001	
	One-way Anova Objective Wellbeing Scores and Neighbourhood	F (6, 299) = 10.817, *p* < 0.001	
3.2.2 Udon Thani Subjective Wellbeing	One-way Anova comparison of SWEMWBS by Neighbourhood	F (6,271) = 2.16, *p* = 0.047	
	One-way Anova comparison of Older (61 + yrs) and younger people’s Perceived Stress Scores	Difference in mean PSS of +1.6. F (3,583) = 5.59, *p* = 0.01	
	One-way Anova comparison of Older (61 + yrs) and younger people’s SWEMWBS	Difference in mean SWEMWBS of -1.9. F (3,583) = 7.35, *p* = 0.01	
3.2.3 Inter-city comparison	One-way Anova comparison of PSS between Nakuru and Udon Thani	F (11,136) = 194.33, *p* < 0.0005	
	One-way Anova comparison of SWEMWBS between Nakuru and Udon Thani	F (11,136) = 1.039, *p* < 0.308	
3.3.1.1 Nakuru Greenspaces	Kruskal-Wallis test of difference in NDVI pixel values by neighbourhood	Pixel Range	Sig
		Values 0–9	0.086
		Values 10–19	0.000
		Values 20–29	0.000
		Values 30–39	0.000
		Values 40–49	0.000
		Values 50–59	0.000
		Values 60–69	0.000
		Values 70–79	0.000
		Values 80–89	0.001
	One-way Anova correlation between NDVI values and neighbourhood affluence	F (11,136) = 1.039, *p* < 0.308	
	Chi2 association between neighbourhood and living within walking distance of a greenspace	χ^2^ (5) = 21.951, *p* = 0.0005	
	Chi2 association between neighbourhood and use of greenspace by surveyed residents	χ2 (5) = 2.980, *p* = 0.703	
	One-way ANOVA comparison of change in SWEMWBS with more than 2 h s time spent in greenspace	F (1,290) = 4.677, *p* = 0.031	
	One-way ANOVA comparison of change in PSS with average greenness of neighbourhoods from NDVI pixel values	F (5,252) = 3.417, *p* = 0.005	
3.3.1.2 Nakuru Public Realm Spaces	Chi2 association between neighbourhood and public space walking distance accessibility	χ2 (5) = 19.189, *p* = 0.002	
	Chi2 association between availability of walking distance public space and use	χ2 (5) = 21.951, *p* = 0.0005	
3.3.1.3 Nakuru Environments Effects on Mood	*t*-test of change in men’s hedonic tone pre- and post- transect walk for those who ended in the public park (pre-mean = 21.41; post-mean = 22.38)	(t (31) = –2.142, *p* = 0.040)	
3.3.1.4 Udon Thani Greenspaces	Kruskal-Wallis test of difference in NDVI pixel values by neighbourhood	Pixel Range	Sig
		Values 0–9	0.154
		Values 10–19	0.013
		Values 20–29	0.001
		Values 30–39	0.102
		Values 40–49	0.080
		Values 50–59	0.002
		Values 60–69	0.002
		Values 70–79	0.034
		Values 80–89	0.999
	Chi2 association between neighbourhood and living within walking distance of a greenspace	χ2 (6) = 103.845, *p* = 0.000	
	Chi2 association between neighbourhood and use of greenspace by surveyed residents	χ2 (6) = 37.056, *p* = 0.000	
3.3.1.5 Udon Thani Public Realm Spaces	Chi2 association between neighbourhood and public space walking distance accessibility	χ2 (6) = 65.664, *p* = 0.000	
3.3.1.6 Udon Thani Environments Effects on Mood	*t*-test of change in all participants hedonic tone pre- and post- transect walk for those who ended in the public park (pre-mean = 23.74; post-mean = 22.32)	t (64) = 3.908, *p* = 0.000	

#### Inter-city Comparison

**FIGURE 2:**
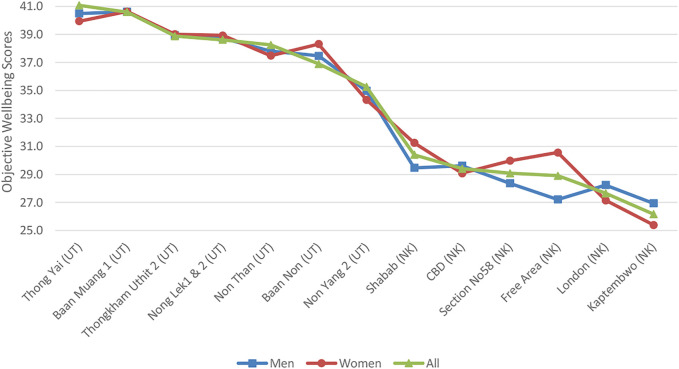
Effects eleven surveyed dimensions of socio-environmental conditions have upon objective wellbeing summed by neighbourhood (**Note:** UT indicates Udon Thani; NK indicates Nakuru)*.*

GDP per capita in 2019 (data.worldbank.org) varied from $7808 for Thailand to $1816 in Kenya indicating significant overall differences in average living standards and economic prosperity between the two countries. Our indicators of socio-economic conditions (relative affluence) confirmed these key differences for our two case study cities. Our Kenyan city has a statistically significant higher number of self-employed and tenants compared to Thailand where more residents were employees and homeowners. Access to basic services were reported as unproblematic across Udon whereas there were significant impacts from lack of access to infrastructure including water in Nakuru. The objective wellbeing scores represent a continuum from the most affluent neighbourhood in Nakuru having similar scores to the least affluent in Udon (see Figure 2).

#### Nakuru Economic and Socio-Environmental Conditions

Employment status (employed versus self-employed) determined which neighbourhood residents can afford to live in. There was no significant difference in objective wellbeing except between the extremes of the best serviced district (Shabab) and the least affluent (semi-informal Kaptembwo). Whilst this indicates similar infrastructure conditions across the majority of Nakuru neighborhoods analyzing by gender reveals significant differences in women’s objective wellbeing scores (whilst men’s do not vary significantly). Access to water, water quality and solid waste pollution were the most important differences in basic services and environmental conditions identified between the semi-informal neighborhoods and planned, more affluent locations.

#### Udon Thani Economic and Socio-Environmental Conditions

Tenancy status varied by neighbourhood indicating differences in home ownership levels across the city. Objective wellbeing varied significantly by neighbourhood. These results confirmed our sample neighborhoods had varying levels of affluence. Of the socio-environmental factors assessed, only traffic congestion was perceived to be having a ‘somewhat negative’ impact on wellbeing.

### Subjective Wellbeing

Our subjective wellbeing metrics varied within our case study cities by neighbourhood and with gender.

#### Nakuru Subjective Wellbeing

Perceived stress tracks with affluence and objective wellbeing metrics and varied significantly between the most (Section 58) and least affluent (Kaptembwo) neighborhoods. This indicates that the absence of basic infrastructure and employment uncertainty has a significant psychological impact on daily life.

**FIGURE 3:**
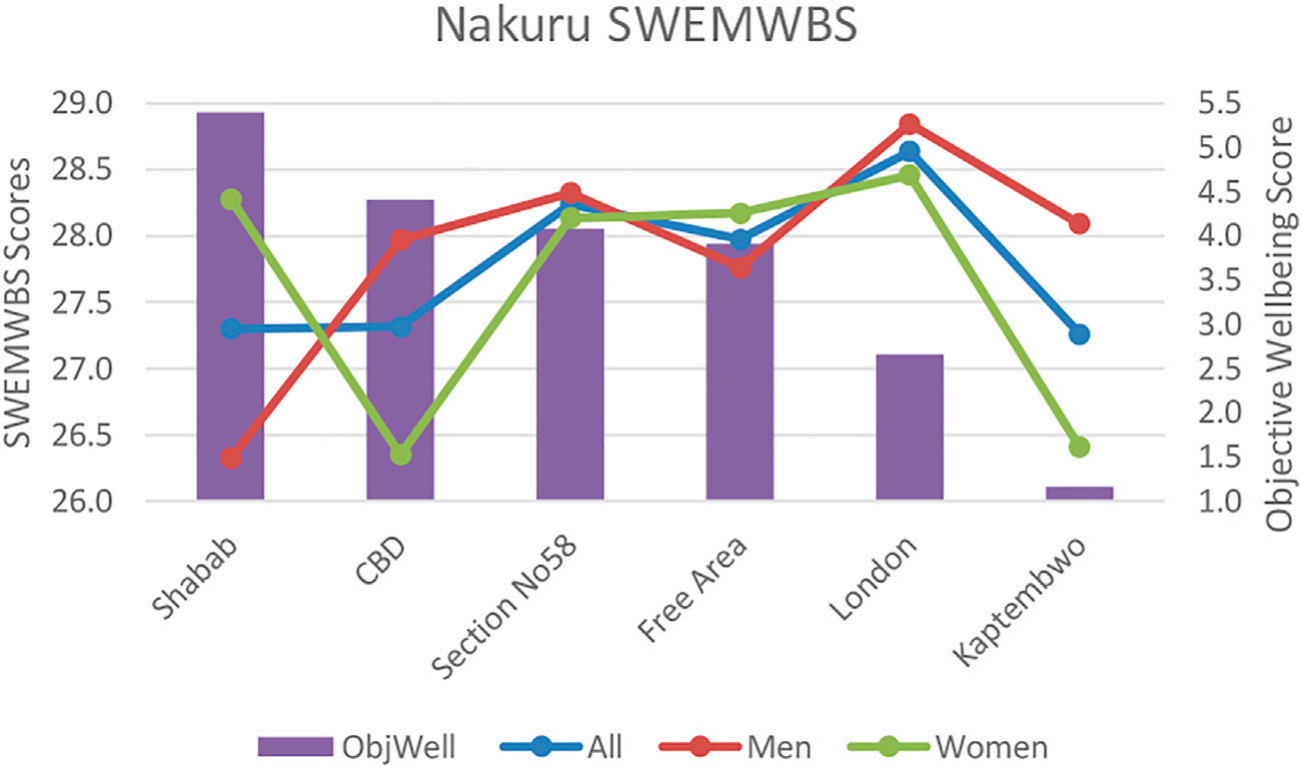
Nakuru Short-Warwick subjective wellbeing scores (SWEMWBS) versus objective wellbeing scores by neighbourhood.

**FIGURE 4:**
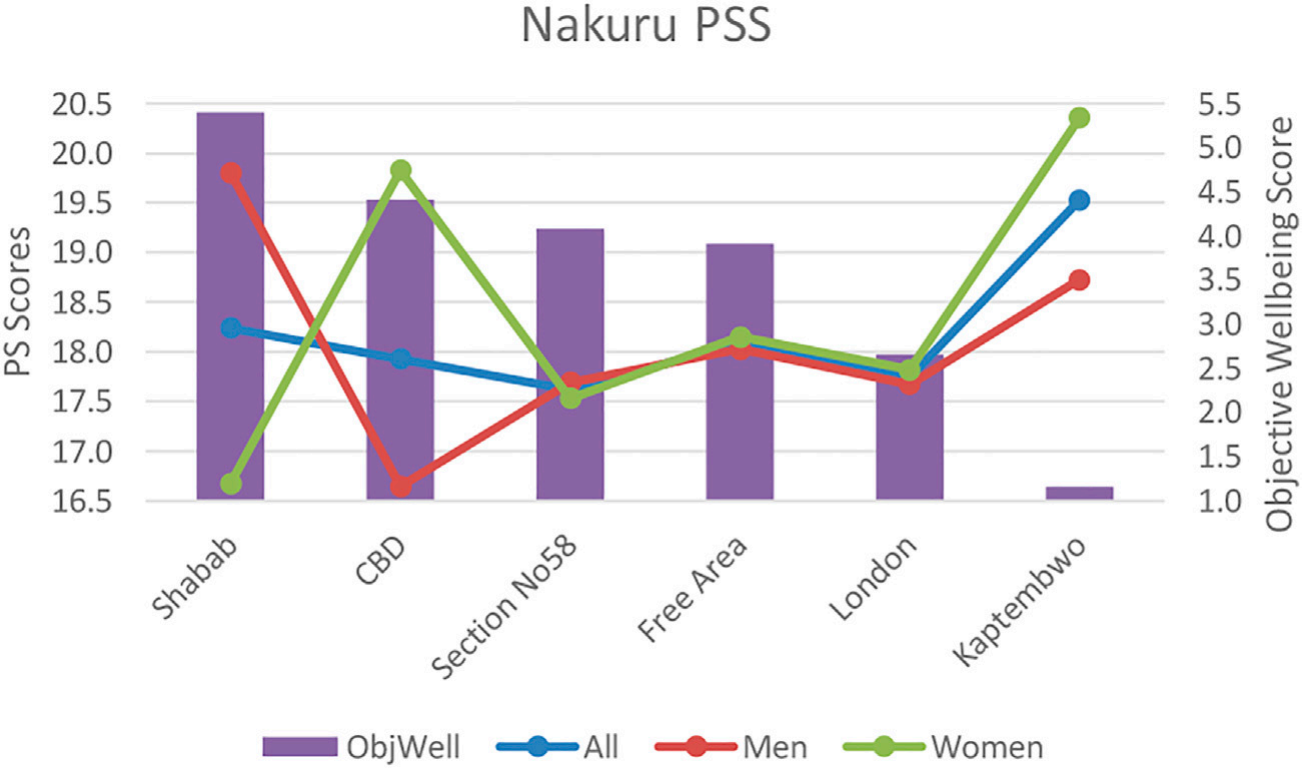
Nakuru perceived stress scale (PSS) scores versus objective wellbeing scores by neighbourhood.

**FIGURE 5:**
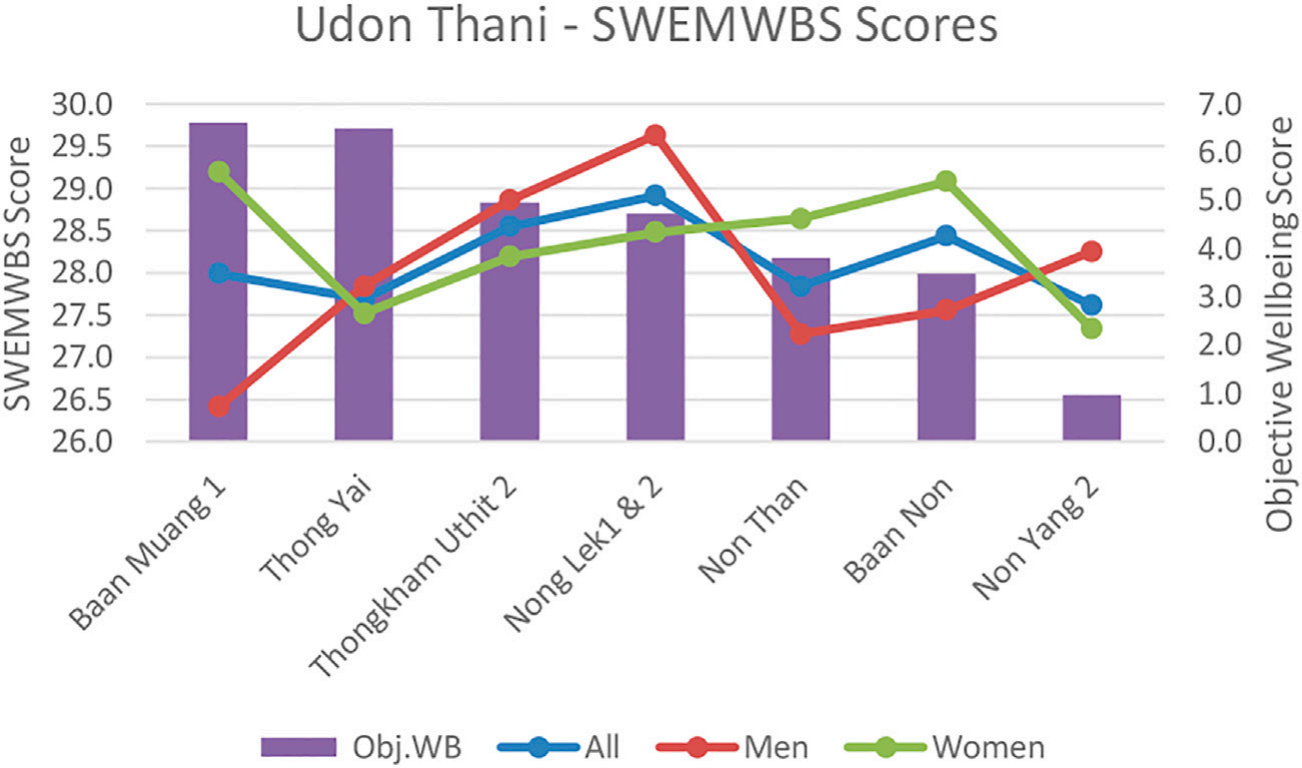
Udon Thani Short-Warwick subjective wellbeing scores (SWEMWBS) versus objective wellbeing scores by neighbourhood.

Our objective wellbeing data indicates that differences in the impacts from social conditions including the incidence of crime and anti-social behaviour between neighborhoods could be underlying factors affecting stress level variations. These take on a gendered dimension with significant differences in women’s PSS between Kaptembwo (large to somewhat negative crime impacts (1.7); somewhat negative anti-social behaviour impacts (2.4) and Shabab (somewhat negative crime (2.26) and anti-social behaviour (2.46) impacts) (see Figure 5). Within neighborhoods, women’s stress was significantly higher than men’s in both the least affluent Kaptembwo but also the more affluent CBD (where crime and behaviour both affect women’s wellbeing more strongly (see supplementary materials: two Assessment of socio-economic conditions) (see Figures 3, 4 below).

Wellbeing scores and stress were not significantly correlated with age.

#### Udon Thani Subjective Wellbeing

**FIGURE 6:**
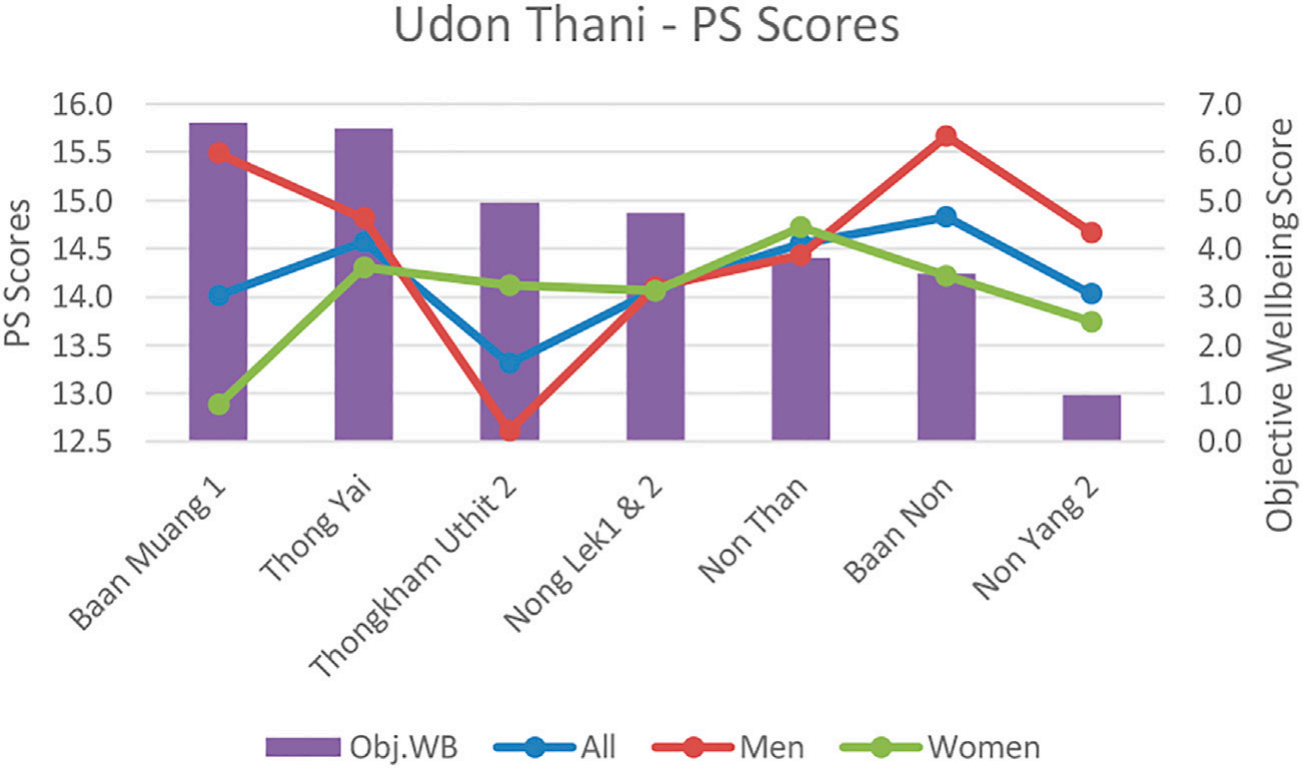
Udon Thani perceived stress scores versus objective wellbeing scores by neighbourhood.

The SWEMWBS are lowest for the extreme’s of high and low objective wellbeing neighborhoods indicating lower overall life satisfaction in these locations (see Figure 5) with the best and worst socio-economic conditions. The surveyed stress scores range from low-to-moderate stress levels and vary independently of affluence indicating other factors are affecting wellbeing beyond employment, tenancy and job type and does not show statistically significant variation by neighbourhood (see Figure 6). We identified that older people (61 + yrs) have lower wellbeing and higher stress levels. Additionally gendered differences emerged between men and women’s stress levels in the affluent (as measured through objective wellbeing) Baan Muang neighbourhood.

#### Inter-city Comparison

Both cities perceived stress results can be characterized as ‘moderate’. Perceived stress in Nakuru (mean score 18.25) was significantly higher than in Udon Thani (mean 14.24), however, subjective wellbeing scores were only marginally different. This highlights that even when urban conditions are a source of persistent stress, longer term personal life satisfaction can remain high.

#### Nakuru Urban Environments

The following sections results present findings relevant to our initial research question of “how is the relationship between subjective wellbeing mediated by the quality of urban environments?”

In our survey, two aspects of the quality of physical environments were considered; availability of greenspace (vegetated parks, sports grounds, temples and woods) and public realm spaces (town square, markets, shopping malls, sports and community centers) both of which can be used for recreation promoting both physical and mental health.

##### Nakuru Greenspaces

**FIGURE 7:**
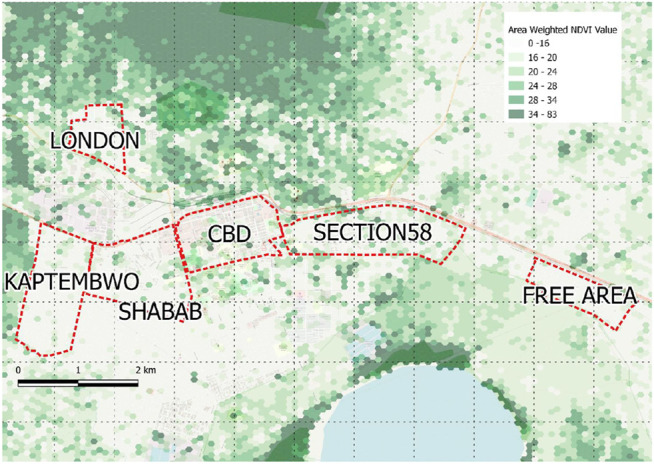
Nakuru Mean NDVI Jan-Dec 2018 **(Landsat Imagery)** [**source:** http://climateengine.org/]. Values have been visualized as mean area weighted NDVI values by 100 m hex grid derived from original 29 m pixels.

Our spatial analysis showed statistically significant differences in NDVI values between neighborhoods (see Figure 7). These were not correlated with affluence indicating some poorer neighborhoods had more greenspace than wealthier locations. Responses to the neighbourhood survey highlighted that availability of walking distance greenspace varied significantly. However, there was no significant relationship between availability and use. These findings indicate that availability of green infrastructure cannot infer usage or accessibility with other factors or preferences either enabling or inhibiting participants use of greenspaces. However, comparing the average greenness of neighborhoods (from the NDVI values) to the PS scores indicated significant stress level reductions. This indicated that more local greenery reduced stress regardless of usage of these environments for recreation.

For those participants who did utilize greenspace, spending greater than 2 h per week in natural environments led to significant improvements in subjective wellbeing (SWEMWBS) (from scores of 26.9 (±4.5) to 28.3 (±5.2)). This 2 h threshold links to recommended “doses” of greenspace use (White et al., 2019) found in other studies from the Global North. Spending longer quantities of time showed no greater improvements with the limited number of respondents exceeding 3 hours having no significant improvements in their subjective wellbeing and stress scores.

##### Nakuru Public Realm Spaces

The survey findings identified a weak but significant association between neighbourhood and walking distance access to public realm spaces. Shabab, the best planned neighbourhood, reported the greatest accessibility (with 76% of respondents reporting they lived within walking distance). The survey also indicated that increased availability of public realm space led to greater use by residents. These results highlight the unequal distribution of public realm assets leading to different opportunities for residents to access sociable community spaces.

##### Nakuru Environments Effects on Mood

The only significant effect of undertaking the transect walk was upon men’s hedonic tone who ended their route in the park. Hedonic tone indicates feelings of happiness or sadness and this result suggests there maybe gendered effects to the benefits from public greenspaces.

##### Udon Thani Greenspaces

**FIGURE 8:**
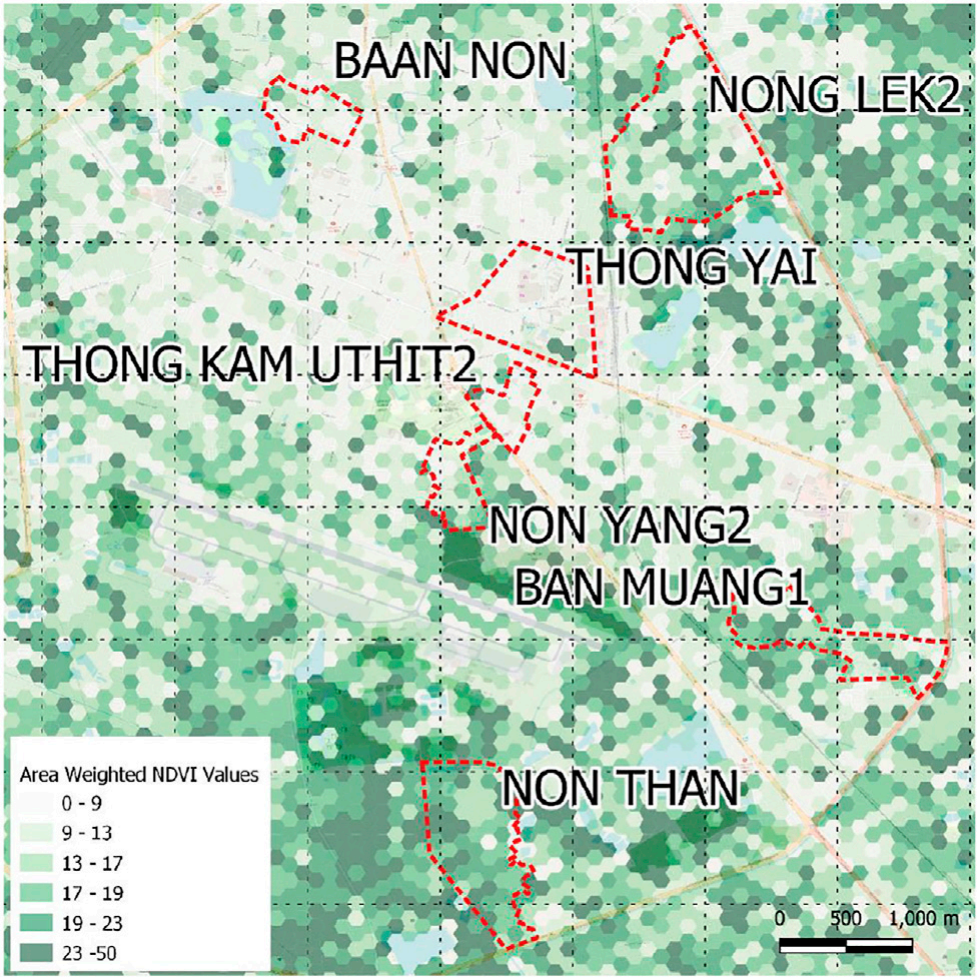
Udon Thani Mean NDVI Jan-Dec 2018 **(Landsat Imagery)** [**source:** http://climateengine.org/] Values have been visualized as mean area weighted NDVI values by 100 m hex grid derived from original 29 m pixels.

Our spatial analysis indicated differences between neighborhoods in terms of their extreme greenness (high NDVI pixel values (50–79) or extreme greyness (low values 10–29) (see Figure 8). These differences manifested in significant differences in residents perceptions of accessibility of walkable distance greenspace. However, the perception of access to greenspace did not always correlate with the measured differences in greenness (NDVI). For example, only 35.6% of Baan Muang one residents indicated that they lived within walking distance of greenspace despite relatively high levels of vegetation (mean NDVI value of 45.05 compared to the highest Non Than with 52.02). Low perceptions of walkable greenspace correlated with significant lower usage of greenspace for recreation.

Use of greenspace did not lead to any significant differences in subjective wellbeing measures (SWEMWBS and PSS). The majority (67.5%) of respondents were making some recreational use of greenspace indicating this behaviour was ubiquitous, however, 65.7% (n = 375) of respondents were spending less than the two 2-h per week threshold. For those who did spend time in greenspace there was no significant relationship or time related benefit on subjective wellbeing or stress scores.

##### Udon Thani Public Realm Spaces

There was a significant difference in the perceived accessibility of public realm spaces by neighbourhood. In general, those neighborhoods on the periphery had less access to public spaces than inner city locations. Approximately 70% of respondents are making use of public realm spaces for recreation, however, greater equality of provision could increase this usage. There was no significant impact on the use or length of time spent in public spaces for recreation on wellbeing or stress.

### Udon Thani Environments Effects on Mood

Looking at the influence of route on the participants in the transect walk, those who began their walk in the park and ended in the market did not see a significant change in hedonic tone (happiness). However, participants who began their transect walk in the market and ended in the park saw a significant decrease (pre-mean = 23.74; post-mean = 22.32) in hedonic tone (indicating increased sadness). These decreases effected both men and women significantly. These results contradict the findings from Nakuru where there were hedonic benefits attributed to greenspace for men.

## Discussion

Our results address how urban quality affects both objective and subjective wellbeing outcomes. The variation in our measured scores between neighborhoods across both cities confirms that our sample sites have a diversity of economic affluence allowing us to usefully compare how objective aspects of local environmental conditions interact with the subjective wellbeing of residents in LMIC settings.

### How Are Objective Aspects of Wellbeing Are Related to Subjective Assessments of Life Satisfaction?

In Kenya, our data indicates how informal and poorly implemented infrastructure has resulted in unequal access to the provision of basic services. Further exploration of these results highlight that poor water access and quality, and solid waste pollution contribute to measurable differences in objective wellbeing impacts between planned and less affluent districts. However, across all neighborhood’s people perceived the limited water access and crime incidence were undermining their wellbeing demonstrating that some challenges are ubiquitous.

In comparison, in Thailand, overall infrastructure and socio-economic conditions were largely un-problematic. However, despite the effects on objective wellbeing being marginal there were a greater number of differences between neighborhoods related to variations in air quality, noise pollution and traffic congestion. This indicates that as relative affluence increases, the significance of marginal inequalities between neighborhoods can become more pronounced. Our Udon Thani data also mirrors findings from other middle income countries (Colombia) (Scopelliti et al., 2016)that mid-affluent communitie’s wellbeing can benefit most from infrastructure availability and environmental improvements (refer to Figures 5, 6).

### How Is the Relationship Between Subjective Wellbeing Mediated by the Quality of Urban Environments?

In Nakuru, subjective wellbeing predominantly lay in the ‘good’ range (scores of 26–28) (Ng Fat et al., 2017) whilst perceived stress fell within the moderate range (scores of 14–26) (State of New Hampshire Employee Assistance Program, 1983). In Udon, wellbeing was “good” to “excellent” (28+) but with between neighbourhoods differences becoming significant. Stress ranged from “moderate” to “low” but did not vary by neighbourhood indicating additional lifestyle factors beyond local environmental conditions were becoming influential on individual mental health. The absence of basic infrastructure (access to water; sanitation) causes significant stress alongside the obvious direct human-health effects (Ritchie and Roser, 2019). The granularity of our findings (at the neighbourhood scale) indicates that unequal access to basic services linked to affluence within LMIC cities significantly affects inequalities in resident’s subjective wellbeing. This contradicts the findings of Kelley and Evans (Kelley and Evans, 2017) who concluded from national survey data that inequality in income distribution boosted wellbeing in low-income countries. Overall, our findings show that city form and quality (both physical infrastructure provision and social interactions) in LMIC cities can have measurable impacts on the subjective wellbeing and stress of residents, potentially undermining their long-term mental health.

Significantly, our Kenya case results demonstrates that residents who make regular use of greenspace (greater than 2-h per week) show benefits to their subjective wellbeing independent of their neighborhoods conditions. This indicates the psychologically restorative benefits of greenspace can offset stress even for those living in informal settlements. In Thailand we did not find associations between wellbeing improvements and greenspace use. This could indicate that other factors influencing ability to spend time in greenspace (e.g. age, employment status) that also affect stress or wellbeing, are masking any benefits of time spent in natural surroundings. The Thai satellite data revealed, whilst there were variations in greenness between the city-centre and peri-urban fringe, most neighbourhoods had significant levels of vegetation. We hypothesize an alternative explanation for these findings that as urban vegetation is more equitably distributed across a city, routinely exposing people to nature, spending time specifically in greenspace has less discernable mental health benefits. The young cohort of Udon Thani transect walk participants indicated a subjective preference for a sociable retail space over a city park again indicating that perhaps greenspace may be less appreciated when it is widely available.

### What Are the Implications for Urban Development to Achieve Equitable Wellbeing Improvements?

Our mixed-method approach highlights the complexity of these inter-relationship; however, they do identify a prioritization for urban planners when considering delivery of life satisfaction improvements. Our cross-city comparison highlights that delivering basic needs infrastructure or services universally must always be the primary city development priority. However, once these services are widely available urban form (distribution of public realm or greenspace) and management (socio-environmental conditions) require greater attention. Wellbeing effects associated with variations in these factors begin to take on a gendered and age dimension independent of neighbourhood affluence (employment and housing status). This conclusion is supported by other studies undertaken in higher income locations (Modai-Snir and van Ham 2018; Patel et al., 2017).

Our findings demonstrate that accessible public realm greenspace and neighbourhood greenery can offset some of the negative impacts on wellbeing of urban living even in challenging environments (socio-economic conditions) including informal settlements counteracting some income related health inequalities (Scopelliti et al., 2016). This supports findings on wellbeing impacts for low-income residents from park use in Indian and Colombian cities (Scopelliti et al., 2016; Ahirrao and Khan, 2021). When combined with the recognized physical health benefits (Siqueira Reis et al., 2013; Canterbury District Health Board, 2016; Adhikari et al., 2019), improved neighbourhood economic prosperity (Ahirrao and Khan, 2021) and co-benefits for active travel (Fluhrer et al., 2021) delivering these features more equitably across cities should be a key consideration for planners.

Our results also highlight that neighbourhood greening needs to be culturally appropriate and relevant for local communities including the urban poor (Ramaswami et al., 2016). This supports call for studies investigating distinct cultural and environmental conditions to make urban greenspace recommendations locally relevant in the Global South (Scopelliti et al., 2016) as the lived experience of residents from African, Asian or Latin American cities can vary distinctly due to factors including interactions of environment and infrastructure (Nagendra et al., 2018). For example, our Thai findings reveal local preferences for incorporating green infrastructure into retail and built public realm spaces to the maximize the distribution of wellbeing and ecosystem service benefits in this urban setting.

### How do Our Findings Compare to Studies From Across Global South Cities?

Our results highlight that distributing greenery throughout cities enables a wider cross-section of residents to enjoy benefits to their underlying wellbeing without needing to spend dedicated time in specific parkland destinations (Cocks et al., 2016; Markevych et al., 2017). This implies cities should incorporate greenspaces through street trees, greened roadside verges, or small-scale pocket parks rather than prioritizing larger but scarcer public parks supporting the findings of (Siqueira Reis et al., 2013). This could begin to counteract the emerging crisis in the rise of non-communicable diseases linked to inactivity and stress identified across South Asia (Adhikari et al., 2019). We add support to the social and spatial justice arguments for widening the distribution of urban greening (Camargo et al., 2017; Rigolon et al., 2018; Zuniga-Teran and Gerlak, 2019; Ahirrao and Khan, 2021) by adding in quantitative evidence on the wellbeing and livability benefits such improvements could bring.

Such distributed greenspace would also ensure equity in other ecosystem service benefits such as urban cooling; shading; biodiversity increases; and surface water flood mitigation (Panagopoulos et al., 2015; Canterbury District Health Board, 2016). Unfortunately, urban greenspace is declining across Global South cities especially rapidly growing secondary cities (Nero, 2017; Adhikari et al., 2019; Fluhrer et al., 2021). As highlighted by (Bai et al., 2018) city planners need greater access to neighbourhood scale data to truly understand the distributional impacts of urban form on resident’s health and city function. For example, internally displaced people residing in Nakuru county have been shown to have poor mental health, quality of life and life satisfaction (Getanda et al., 2015) contrasting with our Nakuru city participants who reported good overall life satisfaction and moderate stress. This demonstrates how high-resolution data is required to identify issues for specific places or population groups understanding local preferences to ensure city developments are appropriate and not merely transferred from different contexts (Nagendra, 2018; Cocks and Shackleton, 2021).

Cross-cutting development issues by their complex nature benefit from an integrated, multi-sector, consultative approach to problem solving if identified solutions are to be resilient (Mitra et al., 2017) in the context of diverse and dynamic city environments. New configurations of actors and collaborations are needed that include vulnerable groups and those typically excluded from city planning (Cinderby et al., 2021; Shackleton et al., 2021). This ambition to make improvements locally relevant (Patel et al., 2017) requires city authorities to plan using participatory co-design approaches that harnesses the collective creativity of people working together in a development process (Lam et al., 2017). Such approaches enable the development of improved shared understandings of complex problems allowing diverse stakeholder to collaborate and agree on locally relevant solutions (McArthur and Robin, 2019). This consensus building aids decision makers identification and delivery of more effective actions (Adelina et al., 2020). These approaches are particularly pertinent when addressing greenspace justice as public institutions typically fund these assets meaning all citizens should enjoy their benefits related to delivery of SDG 11.7 s (Daniel, 2014) ambition to “provide universal access to safe, inclusive and accessible, green and public spaces.”

### Limitations of Findings

The cross-sectional survey data used in this analysis represents a snapshot of conditions at a particular moment. Cities and communities are dynamic - investigating wellbeing’s relationship to changing urban environments would therefore benefit from a long-term longitudinal approach, similar to cohort studies from health sciences. Including a wider range of quantitative data with which to compare subjective wellbeing results and environmental perceptions would also provide a more robust picture of the relationship between people and cities. This could include measuring environmental factors known to affect wellbeing such as air and noise pollution, temperature, and humidity, but also quantitative recording of locally relevant socio-economic conditions such as crime or fluctuations in employment levels. Also improving our understanding in a more nuanced way of the interactions of people and places beyond home neighborhoods would explore temporal and seasonal dimensions. Incorporating more qualitative data from participants would add significant richness and additional context to the findings. Results from a complementary survey undertaken by the paper authors in both case study cities on the cultural ecosystem services that different urban spaces provide addresses this shortfall to a certain extent (Cinderby et al., 2021). We also recognize that focusing on greenness in our analysis lacks inclusivity of other spaces that may be valued within different cultural contexts. We would advocate for a wider definition of beneficial urban infrastructure to include natural (brown-, green-, blue-, and barren spaces) alongside built PRS (indoor and outdoor spaces), and their combinations when looking at the interactions of urban form and wellbeing in the Global South. Finally, this study was only undertaken in two cities; collecting similar data from a wider range of locations would significantly improve the robustness and transferability of our findings allowing a generic set of recommendations for a healthy, liveable city to be identified.

## Conclusion

This study contributes to filling data gaps from LMIC secondary cities on the impacts of urban living on resident’s wellbeing. Our data highlights that delivering basic services to all neighborhoods should be the initial priority. Once these amenities are provided inequalities in the availability of other infrastructure and socio-cultural conditions begin to impact life satisfaction and stress. Our findings indicate that enabling residents to spend 2 hours per week in greenspace may generate similar wellbeing benefits to those identified in European studies. Improving equitable access across cities by dispersing green infrastructure should therefore be a key target for urban planners. Our Thai findings indicate that accessible greened spaces that support social interactions should be the preferred model for implementing these recommendations to support wellbeing for the widest cross-section of city residents.

Rapidly changing cities need to take greater account of the impacts urban form have upon human health and wellbeing. Ensuring equitable access to greenspace entails city authorities prioritize maintaining existing green infrastructure whilst protecting locations that will enable the inclusion of public realm spaces as the urban area expand. Adding improved neighbourhood scale data on human health and wellbeing benefits to the understanding of other ecosystem services provided by urban nature could justify such protection. Our findings indicate that such evidence could counterbalance significant densification pressures driven by cities ambitions to improve efficiency through the conversion of natural spaces into conventional economic assets. Expanding nature provision as cities evolve rather than expensively retrofitting greenspace into built infrastructure is a more cost-effective strategy for LMICs. Further evidence is needed of the financial costs of poor mental health or the economic gains resulting from access to green infrastructure from a wider cross-section of LMIC cities to strengthen these recommendations ensuring they become a key development issue and priority for urban authorities.

## Data Availability

The raw data supporting the conclusions of this article will be made available by the authors, without undue reservation.

## References

[B1] Adelina C., Archer D. Romanus O. J. Opiyo O. (2020). Governing Sustainability in Secondary Cities of the Global South. Stockholm, Sweden: Stockholm Environment Institute.

[B2] Adhikari B. Pokharel S. Mishra S. R. (2019). Shrinking Urban Greenspace and the Rise in Non-communicable Diseases in South Asia: An Urgent Need for an Advocacy. Front. Sustain. Cities 1, 1–5. 10.3389/frsc.2019.00005

[B64] AFDB (2013). African Development Report 2012 Towards Green Growth in Africa. African Development Bank Report.

[B3] Ahirrao P. Khan S. (2021). Assessing Public Open Spaces: A Case of City Nagpur, india. Sustainability 13, 4997. 10.3390/su13094997

[B4] Asian Development Bank (2017). A Region at Risk: The Human Dimensions of Climate Change in Asia and the Pacific. Manila, Philippines. 10.22617/TCS178839-2

[B5] Bai X. Dawson R. J. Ürge-Vorsatz D. Delgado G. C. Salisu Barau A. Dhakal S. (2018). Six Research Priorities for Cities and Climate Change. Nature 555, 23–25. 10.1038/d41586-018-02409-z 29493611

[B6] Berman M. G. Jonides J. Kaplan S. (2008). The Cognitive Benefits of Interacting with Nature. Psychol. Sci. 19, 1207–1212. 10.1111/j.1467-9280.2008.02225.x 19121124

[B7] Bertram C. Rehdanz K. (2015). Preferences for Cultural Urban Ecosystem Services: Comparing Attitudes, Perception, and Use. Ecosystem Serv. 12, 187–199. 10.1016/j.ecoser.2014.12.011

[B65] Bratman G. N. Hamilton J. P. Hahn K. S. Daily G. C. Gross J. J. (2015). Nature Experience Reduces Rumination and Subgenual Prefrontal Cortex Activation. Proc. Natl. Acad. Sci. USA. 112 (28), 8567–8572. 10.1073/pnas.1510459112 26124129 PMC4507237

[B8] Camargo D. M. Ramírez P. C. Fermino R. C. (2017). Individual and Environmental Correlates to Quality of Life in Park Users in Colombia. Int. J. Environ. Res. Public Health 14. 10.3390/ijerph14101250 PMC5664751 29048373

[B9] Canterbury District Health Board. 2016. Associations Between Urban Characteristics and Non-communicable Diseases: Rapid Evidence Review Rapid Evidnce Review. Christchurch, New Zealand: Canterbury District Health Board.

[B10] Cinderby S. de Bruin A. Cambridge H. Muhoza C. Ngabirano A. (2021). Transforming Urban Planning Processes and Outcomes through Creative Methods. Ambio 50, 1018–1034. 10.1007/s13280-020-01436-3 33586051 PMC7882470

[B11] Cocks M. Alexander J. Mogano L. Vetter S. (2016). Ways of Belonging: Meanings of "Nature" AMong Xhosa-Speaking Township Residents in South Africa. J. Ethnobiol. 36, 820–841. 10.2993/0278-0771-36.4.820

[B12] Cocks M. L. Shackleton C. M. (2021). Urban Nature: Enriching Belonging, Wellbeing and Bioculture. Oxford, Taylor and Francis: Routledge.

[B13] Cohen S. Kamarck T. Mermelstein R. (1983). To. Kamarck, and R. Mermelstein.A Global Measure of Perceived Stress. J. Health Soc. Behav. 24, 385–396. 10.2307/2136404 6668417

[B14] Corburn J. (2017). Urban Place and Health Equity: Critical Issues and Practices. Ijerph 14, 1–10. 10.3390/ijerph14020117 PMC5334671 28134756

[B15] Dadvand P. Nieuwenhuijsen M. J. Esnaola M. Forns J. Basagaña X. Alvarez-Pedrerol M. (2015). Green Spaces and Cognitive Development in Primary Schoolchildren. Proc. Natl. Acad. Sci. U S A. 112, 7937–7942. 10.1073/pnas.1503402112 26080420 PMC4491800

[B16] Daniel K. (2014). Goal 11. Make Cities and Human Settlements Inclusive, Safe, Resilient and Sustainable. UN Chronicle. Available at https://www.un.org/en/chronicle/article/goal-11-cities-will-play-important-role-achieving-sdgs .

[B17] Derkzen M. L. Nagendra H. Van Teeffelen A. J. A. Purushotham A. Verburg P. H. (2017). Shifts in Ecosystem Services in Deprived Urban Areas: Understanding People’s Responses and Consequences for Well-Being. Ecology and Society. 22, 1–24. 10.5751/ES-09168-220151

[B18] Ellis P. Roberts M. (2016). Leveraging Urbanization in South Asia: Managing Spatial Transformation for Prosperity and Livability. South Asia Development Matters. Washington, DC: The World Bank. 10.1596/978-1-4648-0662-9

[B19] Elmqvist T. Andersson E. Frantzeskaki N. McPhearson T. Olsson P. Gaffney O. (2019). Sustainability and Resilience for Transformation in the Urban century. Nat. Sustain. 2, 267–273. 10.1038/s41893-019-0250-1

[B20] Fluhrer T. Chapa F. Hack J. (2021). A Methodology for Assessing the Implementation Potential for Retrofitted and Multifunctional Urban green Infrastructure in Public Areas of the Global South. Sustainability 13, 1–25. 10.3390/su13010384

[B21] Getanda E. M. Papadopoulos C. Evans H. (2015). The Mental Health, Quality of Life and Life Satisfaction of Internally Displaced Persons Living in Nakuru County, Kenya. BMC Public Health 15, 755. 10.1186/s12889-015-2085-7 26246147 PMC4527222

[B22] Grimm N. B. Faeth S. H. Golubiewski N. E. Redman C. L. Wu J. Bai X. (2008). Global Change and the Ecology of Cities. Science 319, 756–760. 10.1126/science.1150195 18258902

[B23] Hansen R. Olafsson A. S. van der Jagt A. P. N. Rall E. Pauleit S. (2019). Planning Multifunctional green Infrastructure for Compact Cities: What Is the State of Practice? Ecol. Indicators 96, 99–110. 10.1016/j.ecolind.2017.09.042

[B24] Kelley J. Evans M. D. R. (2017). The New Income Inequality and Well-Being Paradigm: Inequality Has No Effect on Happiness in Rich Nations and normal Times, Varied Effects in Extraordinary Circumstances, Increases Happiness in Poor Nations, and Interacts with Individual's Perceptions, Attitudes, Politics, and Expectations for the Future. Soc. Sci. Res. 62, 39–74. 10.1016/j.ssresearch.2016.12.007 28126114

[B25] Koushede V. Lasgaard M. Hinrichsen C. Meilstrup C. Nielsen L. Rayce S. B. (2019). Measuring Mental Well-Being in Denmark: Validation of the Original and Short Version of the Warwick-Edinburgh Mental Well-Being Scale (WEMWBS and SWEMWBS) and Cross-Cultural Comparison across Four European Settings. Psychiatry Res. 271, 502–509. 10.1016/j.psychres.2018.12.003 30551082

[B26] Lam B. Zamenopoulos T. Kelemen M. Hoo Na J. (2017). Unearth Hidden Assets through Community Co-design and Co-production. Des. J. 20, S3601–S3610. 10.1080/14606925.2017.1352863

[B63] Leach J. M. Lee S. E. Braithwaite P. A. Bouch C. J. Grayson N. Rogers C. D. F. (2014). What Makes a City Liveable? Implications for Next-Generation Infrastructure Services. 10.14453/isngi2013.proc.29

[B27] Markevych I. Schoierer J. Hartig T. Chudnovsky A. Hystad P. Dzhambov A. M. (2017). Exploring Pathways Linking Greenspace to Health: Theoretical and Methodological Guidance. Environ. Res. 158, 301–317. 10.1016/j.envres.2017.06.028 28672128

[B28] McArthur J. Robin E. (2019). Victims of Their Own (Definition of) success: Urban Discourse and Expert Knowledge Production in the Liveable City. Urban Stud. 56, 1711–1728. 10.1177/0042098018804759

[B29] McFall S. L. Garrington C. (2011). Early Findings From the First Wave of the UK’s Household Longitudinal Study. Colchester: Institute for Social and Economic Research, University of Essex

[B30] McPhearson T. Pickett S. T. A. Grimm N. B. Niemelä J. Alberti M. Elmqvist T. (2016). Advancing Urban Ecology toward a Science of Cities. BioScience 66, 198–212. 10.1093/biosci/biw002

[B31] Mitra S. Mulligan J. Schilling J. Harper J. Vivekananda J. Krause L. (2017). Developing Risk or Resilience? Effects of Slum Upgrading on the Social Contract and Social Cohesion in Kibera, Nairobi. Environ. Urbanization 29, 103–122. 10.1177/0956247816689218

[B67] Modai-Snir T. van Ham M. (2018). Neighbourhood Change and Spatial Polarization: The Roles of Increasing Inequality and Divergent Urban Development. Cities 82, 108–118. 10.1016/j.cities.2018.05.009

[B32] Nagendra H. (2018). The Global South Is Rich in Sustainability Lessons that Students Deserve to Hear. Nature 557, 485–488. 10.1038/d41586-018-05210-0 29789749

[B33] Nagendra H. Bai X. Brondizio E. S. Lwasa S. (2018). The Urban South and the Predicament of Global Sustainability. Nat. Sustain. 1, 341–349. 10.1038/s41893-018-0101-5

[B34] Neale C. Besa M. C. Dickin S. Hongsathavij V. Kuldna P. Muhoza C. (2019). Comparing Health, Stress, Wellbeing and Greenspace across Six Cities in Three Continents. Cities & Health 4, 290–302. 10.1080/23748834.2019.1696648

[B35] Nero B. F. (2017). Urban green Space Dynamics and Socio-Environmental Inequity: Multi-Resolution and Spatiotemporal Data Analysis of Kumasi, ghana. Int. J. Remote Sensing 38, 6993–7020. 10.1080/01431161.2017.1370152

[B36] Ng Fat L. Scholes S. Boniface S. Mindell J. Stewart-Brown S. (2017). Evaluating and Establishing National Norms for Mental Wellbeing Using the Short Warwick-Edinburgh Mental Well-Being Scale (SWEMWBS): Findings from the Health Survey for England. Qual. Life Res. 26, 1129–1144. 10.1007/s11136-016-1454-8 27853963 PMC5376387

[B37] Nordbakke S. Schwanen T. (2013). Well-being and Mobility: A Theoretical Framework and Literature Review Focusing on Older People. Mobilities 9, 104–129. 10.1080/17450101.2013.784542

[B39] Panagopoulos T. González Duque J. A. Bostenaru Dan M. (2015). Urban Planning with Respect to Environmental Quality and Human Well-Being. Environ. Pollut. 208, 137–144. 10.1016/j.envpol.2015.07.038 26243477

[B40] Patel Z. Greyling S. Simon D. Arfvidsson H. Moodley N. Primo N. (2017). Local Responses to Global Sustainability Agendas: Learning from Experimenting with the Urban Sustainable Development Goal in Cape Town. Sustain. Sci. 12, 785–797. 10.1007/s11625-017-0500-y 30147761 PMC6086247

[B41] Pauleit S. Vasquez A. Maruthaveeran S. Liu L. Cilliers S. (2021). “Urban Green Infrastructure in the Global South,” in Urban Ecology in the Global South (Cham: Springer Nature). 10.1007/978-3-030-67650-6_5

[B42] Ramaswami A. Russell A. G. Culligan P. J. Sharma K. R. Kumar E. (2016). Meta-principles for Developing Smart, Sustainable, and Healthy Cities. Science 352, 940–943. 10.1126/science.aaf7160 27199418

[B43] Rigolon A. Browning M. Lee K. Shin S. (2018). Access to Urban Green Space in Cities of the Global South: A Systematic Literature Review. Urban Sci. 2, 67. 10.3390/urbansci2030067

[B44] Ritchie H. Roser M. (2019). “Sanitation,” in Our World in Data. Available at: https://ourworldindata.org/sanitation#

[B45] Roe J. J. Thompson C. W. Aspinall P. A. Brewer M. J. Duff E. I. Miller D. (2013). Green Space and Stress: Evidence from Cortisol Measures in Deprived Urban Communities. Int. J. Environ. Res. Public Health 10, 4086–4103. 10.3390/ijerph10094086 24002726 PMC3799530

[B46] Scopelliti M. Carrus G. Adinolfi C. Suarez G. Colangelo G. Lafortezza R. (2016). Staying in Touch with Nature and Well-Being in Different Income Groups: The Experience of Urban parks in Bogotá. Landscape Urban Plann. 148, 139–148. 10.1016/j.landurbplan.2015.11.002

[B47] Shackleton C. M. Cilliers S. S. Davoren E. du Toit M. J. (2021). Urban Ecology in the Global South. Springer International Publishing. Cities and Nature.

[B48] Siqueira Reis R. Hino A. A. Ricardo Rech C. Kerr J. Curi Hallal P. (2013). Walkability and Physical Activity: Findings from Curitiba, Brazil. Am. J. Prev. Med. 45, 269–275. 10.1016/j.amepre.2013.04.020 23953352 PMC3748398

[B49] Smit W. (2018). Urban Governance in Africa: An Overview. Part 2-Urban Governance 10, 55–77. 10.4000/poldev.2637

[B50] Soja E. W. (2010). Seeking Spatial Justice. Minneapolis, MN: University of Minnesota Press.

[B51] State of New Hampshire Employee Assistance Program (1983). Perceived Stress Scale Score Cut Off. State of New Hampshire Employee Assistance Program. Concord: State of New Hampshire. 10.1037/t02889-000

[B52] Stewart-Brown S. Tennant A. Tennant R. Platt S. Parkinson J. Weich S. (2009). Internal Construct Validity of the Warwick-Edinburgh Mental Well-Being Scale (WEMWBS): A Rasch Analysis Using Data from the Scottish Health Education Population Survey. Health Qual. Life Outcomes 7, 1–8. 10.1186/1477-7525-7-15 19228398 PMC2669062

[B53] Taylor L. Hochuli D. F. (2017). Defining Greenspace: Multiple Uses across Multiple Disciplines. Landscape Urban Plann. 158, 25–38. 10.1016/j.landurbplan.2016.09.024

[B54] Thomson D. R. Linard C. Vanhuysse S. Steele J. E. Shimoni M. Siri J. (2019). Extending Data for Urban Health Decision-Making: a Menu of New and Potential Neighborhood-Level Health Determinants Datasets in LMICs. J. Urban Healthjournal Urban Health 96, 514–536. 10.1007/s11524-019-00363-3 PMC6677870 31214975

[B55] UN-Habitat (2015). Habitat III Issue Paper 22: Informal Settlements. New York, NY: UN-Habitat, 2015. 10.18772/22014107656.12

[B56] Habitat III Secretariat-United Nations (2017). New Urban Agenda. Geneva: United Nations.

[B57] United Nations: Department of Economic and Social Affairs Population Division (2019). World Urbanization Prospects: The 2018 Revision. New York: ST/ESA/SER.A/420. 10.4054/demres.2005.12.9

[B58] Western M. Tomaszewski W. (2016). Subjective Wellbeing, Objective Wellbeing and Inequality in Australia. PLoS ONE 11, e0163345–20. 10.1371/journal.pone.0163345 27695042 PMC5047468

[B66] White M. P. Alcock I. Grellier J. Wheeler B. W. Hartig T. Warber S. L. (2019). Spending at Least 120 Minutes a Week in Nature is Associated With Good Health and Wellbeing. Sci. Rep. 9 (1), 1–11. 10.1038/s41598-019-44097-3 31197192 PMC6565732

[B59] WHO (2016). Healthy Cities: - Good Health Is Good Politics: Toolkit for Local Governments to Support Healthy Urban Development.

[B60] Winkler T. (2012). Between Economic Efficacy and Social justice: Exposing the Ethico-Politics of Planning. Cities 29, 166–173. 10.1016/j.cities.2011.11.014

[B61] World Health Organization (WHO) (2016). Healthy Cities-Good Health Is Good Politics: Toolkit for Local Governments to Support Healthy Urban Development. Geneva: WHO Press. Available at: http://www.euro.who.int/en/health-topics/environment-and-health/urban-health/activities/healthy-cities

[B62] Zuniga-Teran A. Gerlak A. (2019). A Multidisciplinary Approach to Analyzing Questions of justice Issues in Urban Greenspace. Sustainability 11, 1–22. 10.3390/su11113055

