# Corrigendum: Assessing Inequalities in Wellbeing at a Neighbourhood Scale in Low-Middle-Income-Country Secondary Cities and Their Implications for Long-Term Livability

**DOI:** 10.3389/fsoc.2022.856609

**Published:** 2022-02-15

**Authors:** Steve Cinderby, Diane Archer, Vishal K. Mehta, Chris Neale, Romanus Opiyo, Rachel M. Pateman, Cassilde Muhoza, Charrlotte Adelina, Heidi Tuhkanen

**Affiliations:** ^1^Stockholm Environment Institute, Environment and Geography Department, University of York, York, United Kingdom; ^2^Stockholm Environment Institute, Asia Centre, Bangkok, Thailand; ^3^Stockholm Environment Institute, US Centre, Davis, CA, United States; ^4^Department of Psychology, University of Huddersfield, Huddersfield, United Kingdom; ^5^Stockholm Environment Institute, Africa Centre, Nairobi, Kenya; ^6^Stockholm Environment Institute, Tallinn Centre, Tallinn, Estonia; ^7^Faculty of Biological and Environmental Sciences, University of Helsinki, Helsinki, Finland

**Keywords:** wellbeing, equity, urban, planning, livability, greenspace, global south

An author name was incorrectly spelled as Heidi Tukhanen. The correct spelling is Heidi Tuhkanen.

In the published article, there was an error regarding the affiliation(s) for Heidi Tuhkanen. As well as having affiliation(s) 6, they should also have Faculty of Biological and Environmental Sciences, University of Helsinki, Helsinki, Finland.

The authors apologize for this error and state that this does not change the scientific conclusions of the article in any way. The original article has been updated.

## Publisher's Note

All claims expressed in this article are solely those of the authors and do not necessarily represent those of their affiliated organizations, or those of the publisher, the editors and the reviewers. Any product that may be evaluated in this article, or claim that may be made by its manufacturer, is not guaranteed or endorsed by the publisher.

