# Supplementary material for: Assessing Inequalities in Wellbeing at a Neighbourhood Scale in Low-Middle-Income-Country Secondary Cities and Their Implications for Long-Term Livability
Source: Front Sociol. 2021 Nov 8;6:729453. doi: 10.3389/fsoc.2021.729453 (PMC8651492; doi:10.3389/fsoc.2021.729453)
Supplement: Supplementary file 1 [file Table1.DOCX]

Supplementary Material

# Participant Numbers

## Wellbeing Surveys

### Nakuru Participant Numbers

| **Neighbourhood** | **All** | **Men** | **Women** |
| --- | --- | --- | --- |
| ***CBD*** | 57 | 34 | 23 |
| ***Free Area*** | 78 | 38 | 40 |
| ***Kaptembwo*** | 130 | 66 | 64 |
| ***London*** | 113 | 52 | 61 |
| ***Section No58*** | 100 | 55 | 45 |
| ***Shabab*** | 50 | 25 | 25 |
| ***Total*** | 528 | 270 | 258 |

### Udon Thani Participant Numbers

| **Neighbourhood** | **All** | **Men** | **Women** |
| --- | --- | --- | --- |
| ***Baan Non*** | 64 | 27 | 37 |
| ***Thong Yai*** | 136 | 70 | 66 |
| ***Baan Muang 1*** | 90 | 39 | 51 |
| ***Thongkham Uthit 2*** | 91 | 49 | 42 |
| ***Non Yang 2*** | 39 | 12 | 27 |
| ***Nong Lek1 & 2*** | 80 | 30 | 50 |
| ***Non Than*** | 87 | 51 | 36 |
| ***Total*** | 587 | 278 | 309 |

## Participants and transect walks

Transect walks were undertaken between a public realm greenspace (park) through increasingly busy urban spaces to a retail market or shopping mall. Participants were stratified into two groups who independently undertook the walk in opposing directions. In Udon thani the route was 1.1km whilst in Nakuru it was 717m. Walks were undertaken early or late in the day to avoid excessive heat.

A mix of approximately evenly balanced male and female adult participants were recruited aged between 18 and 30 years who self-reported no underlying health conditions or mobility problems. In Udon thani 115 participants were recruited with no underlying self-reported health concerns or mobility issues (58 women – aged between 18 & 30 – Mn age 24.12; 57 men – aged between 19 & 30 – Mn 24.67). In Nakuru 122 participants were recruited with no underlying self-reported health concerns or mobility issues (58 women – aged between 18 & 30 – Mn age 24.12; 64 men – aged between 19 & 30 – Mn 24./9).

# Assessment of socio-economic conditions

## Homeownership & tenure

Participants were asked whether they were homeowners, tenants (which included housing supplied through employment) or other tenancy status. The percentage of the respondents in each class was generated. The percentage in each tenancy class were ranked by district and the rank value of homeownership used to generate the tenure status.

## Employment status

Participants were asked whether they were employed, self-employed or unemployed (this includes retired). The percentage of the respondents in each class was generated. The percentage in each employment class were ranked by district and the Mn rank value of employed and self-employed was calculated.

## Job description

The participants job descriptions were classified into professional (class 1) (e.g. teacher or pharmacist), semi-professional (class 2) (business owners or managers), manual labour and semi-skilled (class 3) (e.g. welder or shop-assistant), and unemployed (class 4) (including students and retired). The weighted Mn of the job description was then calculated by district. The inverse rank of this score (higher rank equates to greater prevalence of professional employment) was taken to indicate the Mn job class of the neighbourhood.

### Affluence class calculation

The Mn sum of the ranked values for homeownership, employment and job description was calculated to identify the relative affluence of each sampled neighbourhood in each study area.

## Table 1: Objective wellbeing scores by neighbourhood broken down by gender.

| Location |  |  | Crime impact on health and well being | Poor water quality impact on health and well being | Limited access to water impact on health and well being | Noise pollution impact on health and wellbeing | Air pollution impact on health and wellbeing | Solid waste pollution impact | Traffic congestion impact on health and wellbeing | Antisocial behaviour impact on health and wellbeing | Lack of public spaces impact on health and wellbeing | Access to public transport impact to health and well being | Lack of access to social services impact on health and wellbeing |
| --- | --- | --- | --- | --- | --- | --- | --- | --- | --- | --- | --- | --- | --- |
| CBD | Male | N | 32 | 32 | 32 | 32 | 32 | 32 | 32 | 32 | 32 | 32 | 32 |
|  |  | Mn | 2.38 | 2.47 | 2.22 | 3.06 | 2.84 | 2.44 | 2.59 | 2.75 | 2.88 | 3.03 | 2.97 |
|  |  | SD | 1.31 | 1.34 | 1.18 | 1.13 | 1.19 | 1.27 | 1.16 | 1.24 | 1.07 | 1.12 | 1.06 |
|  | Fem-ale | N | 22 | 22 | 22 | 22 | 22 | 22 | 22 | 22 | 22 | 22 | 22 |
|  |  | Mn | 2.14 | 2.32 | 2.41 | 2.73 | 2.64 | 2.55 | 2.91 | 2.50 | 2.73 | 3.23 | 2.95 |
|  |  | SD | 1.28 | 1.36 | 1.18 | 1.32 | 1.18 | 1.37 | 1.23 | 1.19 | 1.12 | 1.02 | 1.05 |
|  | Total | N | 54 | 54 | 54 | 54 | 54 | 54 | 54 | 54 | 54 | 54 | 54 |
|  |  | Mn | 2.28 | 2.41 | 2.30 | 2.93 | 2.76 | 2.48 | 2.72 | 2.65 | 2.81 | 3.11 | 2.96 |
|  |  | SD | 1.29 | 1.34 | 1.18 | 1.21 | 1.18 | 1.30 | 1.19 | 1.22 | 1.08 | 1.08 | 1.05 |
| Free Area | Male | N | 38 | 38 | 38 | 38 | 38 | 38 | 38 | 38 | 38 | 38 | 38 |
|  |  | Mn | 1.61 | 2.18 | 1.95 | 2.84 | 2.39 | 2.58 | 2.82 | 2.55 | 2.39 | 3.34 | 2.55 |
|  |  | SD | 0.97 | 1.31 | 1.09 | 1.20 | 1.20 | 1.13 | 1.11 | 1.11 | 1.10 | 0.78 | 1.20 |
|  | Fem-ale | N | 40 | 40 | 40 | 40 | 40 | 40 | 39 | 40 | 40 | 40 | 40 |
|  |  | Mn | 2.18 | 2.55 | 2.15 | 2.90 | 2.75 | 2.70 | 3.51 | 2.73 | 2.83 | 3.58 | 2.73 |
|  |  | SD | 1.28 | 1.22 | 1.19 | 1.24 | 1.28 | 1.24 | 0.82 | 1.13 | 1.22 | 0.64 | 1.28 |
|  | Total | N | 78 | 78 | 78 | 78 | 78 | 78 | 77 | 78 | 78 | 78 | 78 |
|  |  | Mn | 1.90 | 2.37 | 2.05 | 2.87 | 2.58 | 2.64 | 3.17 | 2.64 | 2.62 | 3.46 | 2.64 |
|  |  | SD | 1.17 | 1.27 | 1.14 | 1.21 | 1.24 | 1.18 | 1.03 | 1.12 | 1.18 | 0.72 | 1.24 |
| Kaptem-bwo | Male | N | 63 | 65 | 65 | 65 | 65 | 65 | 63 | 65 | 65 | 65 | 65 |
|  |  | Mn | 1.63 | 2.15 | 1.98 | 2.31 | 2.17 | 1.97 | 2.98 | 3.02 | 2.75 | 3.14 | 2.74 |
|  |  | SD | 1.07 | 1.19 | 1.07 | 1.12 | 1.13 | 1.06 | 1.17 | 1.22 | 1.31 | 1.10 | 1.29 |
|  | Fem-ale | N | 63 | 63 | 63 | 62 | 63 | 62 | 63 | 63 | 63 | 63 | 63 |
|  |  | Mn | 1.70 | 2.22 | 1.70 | 2.31 | 2.27 | 2.06 | 3.05 | 2.46 | 2.52 | 2.95 | 2.35 |
|  |  | SD | 1.14 | 1.24 | 0.93 | 1.22 | 1.27 | 1.21 | 1.21 | 1.28 | 1.24 | 1.24 | 1.30 |
|  | Total | N | 126 | 128 | 128 | 127 | 128 | 127 | 126 | 128 | 128 | 128 | 128 |
|  |  | Mn | 1.67 | 2.19 | 1.84 | 2.31 | 2.22 | 2.02 | 3.02 | 2.74 | 2.64 | 3.05 | 2.55 |
|  |  | SD | 1.10 | 1.21 | 1.01 | 1.17 | 1.20 | 1.13 | 1.19 | 1.28 | 1.28 | 1.17 | 1.30 |
| London | Male | N | 52 | 52 | 52 | 52 | 52 | 52 | 52 | 52 | 52 | 52 | 52 |
|  |  | Mn | 1.85 | 2.31 | 2.04 | 2.56 | 2.58 | 2.33 | 3.37 | 2.71 | 2.46 | 3.44 | 2.62 |
|  |  | SD | 1.06 | 1.28 | 1.05 | 1.29 | 1.26 | 1.25 | 0.89 | 1.21 | 1.13 | 0.80 | 1.27 |
|  | Fem-ale | N | 61 | 61 | 61 | 61 | 61 | 61 | 60 | 61 | 61 | 61 | 61 |
|  |  | Mn | 1.95 | 2.10 | 1.95 | 2.61 | 2.23 | 2.23 | 3.12 | 2.75 | 2.48 | 3.20 | 2.59 |
|  |  | SD | 1.12 | 1.19 | 1.09 | 1.20 | 1.20 | 1.31 | 1.12 | 1.26 | 1.15 | 1.01 | 1.33 |
|  | Total | N | 113 | 113 | 113 | 113 | 113 | 113 | 112 | 113 | 113 | 113 | 113 |
|  |  | Mn | 1.90 | 2.19 | 1.99 | 2.58 | 2.39 | 2.27 | 3.23 | 2.73 | 2.47 | 3.31 | 2.60 |
|  |  | SD | 1.09 | 1.23 | 1.06 | 1.24 | 1.24 | 1.28 | 1.02 | 1.23 | 1.13 | 0.93 | 1.30 |
| Section No58 | Male | N | 54 | 54 | 54 | 54 | 54 | 54 | 54 | 54 | 54 | 54 | 54 |
|  |  | Mn | 2 | 2.56 | 2.24 | 2.83 | 2.85 | 2.59 | 2.76 | 2.41 | 2.57 | 3.19 | 2.37 |
|  |  | SD | 1.06 | 1.13 | 0.99 | 1.04 | 1.05 | 1.06 | 1.21 | 1.04 | 1.13 | 0.99 | 1.17 |
|  | Fem-ale | N | 43 | 43 | 43 | 43 | 43 | 43 | 43 | 43 | 43 | 43 | 43 |
|  |  | Mn | 2.26 | 2.44 | 2.47 | 2.72 | 2.88 | 2.65 | 3 | 2.72 | 2.91 | 3.09 | 2.84 |
|  |  | SD | 1.14 | 1.22 | 1.14 | 1.14 | 1.10 | 1.13 | 1.15 | 1.14 | 1.13 | 0.97 | 1.04 |
|  | Total | N | 97 | 97 | 97 | 97 | 97 | 97 | 97 | 97 | 97 | 97 | 97 |
|  |  | Mn | 2.11 | 2.51 | 2.34 | 2.78 | 2.87 | 2.62 | 2.87 | 2.55 | 2.72 | 3.14 | 2.58 |
|  |  | SD | 1.10 | 1.16 | 1.06 | 1.08 | 1.07 | 1.08 | 1.19 | 1.09 | 1.13 | 0.98 | 1.14 |
| Shabab | Male | N | 25 | 25 | 25 | 25 | 25 | 25 | 25 | 25 | 25 | 25 | 25 |
|  |  | Mn | 2.16 | 2.60 | 2.12 | 2.72 | 2.56 | 2.84 | 2.92 | 2.76 | 2.88 | 3.08 | 2.84 |
|  |  | SD | 1.21 | 1.22 | 0.97 | 1.02 | 1.16 | 1.11 | 1.04 | 1.09 | 1.05 | 1 | 1.03 |
|  | Fem-ale | N | 23 | 23 | 23 | 23 | 23 | 23 | 23 | 23 | 23 | 23 | 23 |
|  |  | Mn | 2.26 | 2.74 | 2.61 | 3 | 2.74 | 2.91 | 2.87 | 2.78 | 3 | 3.13 | 3.22 |
|  |  | SD | 1.14 | 1.36 | 1.12 | 1.09 | 1.25 | 1.08 | 1.10 | 1.24 | 0.95 | 1.01 | 1.04 |
|  | Total | N | 48 | 48 | 48 | 48 | 48 | 48 | 48 | 48 | 48 | 48 | 48 |
|  |  | Mn | 2.21 | 2.67 | 2.35 | 2.85 | 2.65 | 2.88 | 2.90 | 2.77 | 2.94 | 3.10 | 3.02 |
|  |  | SD | 1.17 | 1.28 | 1.06 | 1.05 | 1.19 | 1.08 | 1.06 | 1.15 | 1 | 0.99 | 1.04 |
| Total | Male | N | 264 | 266 | 266 | 266 | 266 | 266 | 264 | 266 | 266 | 266 | 266 |
|  |  | Mn | 1.89 | 2.35 | 2.08 | 2.67 | 2.54 | 2.39 | 2.94 | 2.71 | 2.64 | 3.22 | 2.65 |
|  |  | SD | 1.12 | 1.24 | 1.05 | 1.16 | 1.18 | 1.16 | 1.12 | 1.17 | 1.16 | 0.98 | 1.20 |
|  | Fem-ale | N | 252 | 252 | 252 | 251 | 252 | 251 | 250 | 252 | 252 | 252 | 252 |
|  |  | Mn | 2.02 | 2.34 | 2.11 | 2.65 | 2.52 | 2.43 | 3.10 | 2.65 | 2.69 | 3.17 | 2.68 |
|  |  | SD | 1.18 | 1.25 | 1.12 | 1.21 | 1.24 | 1.25 | 1.12 | 1.21 | 1.17 | 1.03 | 1.24 |
|  | Total | N | 516 | 518 | 518 | 517 | 518 | 517 | 514 | 518 | 518 | 518 | 518 |
|  |  | Mn | 1.95 | 2.34 | 2.09 | 2.66 | 2.53 | 2.41 | 3.02 | 2.68 | 2.66 | 3.20 | 2.67 |
|  |  | SD | 1.15 | 1.24 | 1.09 | 1.18 | 1.21 | 1.21 | 1.13 | 1.19 | 1.16 | 1 | 1.22 |
| Neighbourhood |  |  | Poor water quality impact on health and well being | Limited access to water impact on health and wellbeing | Noise pollution impact on health and wellbeing | Air pollution impact on health and well being | Solid waste pollution impact on health and well being | Traffic congestion impact on health and wellbeing | Antisocial behaviours impact on health and well being | Crime impact on health and wellbeing | Lack of public space impact | Public transport access impact on health and wellbeing | Social services impact on health and wellbeing |
| Baan Non | Male | N | 27 | 27 | 27 | 27 | 27 | 26 | 27 | 27 | 27 | 27 | 27 |
|  |  | Mn | 3.59 | 3.74 | 2.89 | 3.41 | 3.56 | 2.54 | 3.56 | 3.85 | 3.81 | 3.56 | 3.93 |
|  |  | SD | 0.57 | 0.45 | 0.97 | 0.75 | 0.75 | 0.95 | 0.64 | 0.36 | 0.56 | 0.89 | 0.27 |
|  | Fem-ale | N | 37 | 37 | 37 | 37 | 37 | 37 | 37 | 37 | 37 | 37 | 37 |
|  |  | Mn | 3.22 | 3.43 | 2.76 | 3.30 | 3.19 | 2.32 | 3.43 | 3.81 | 3.86 | 3.68 | 3.89 |
|  |  | SD | 0.82 | 0.65 | 0.76 | 0.74 | 0.88 | 1.25 | 0.73 | 0.40 | 0.35 | 0.67 | 0.39 |
|  | Total | N | 64 | 64 | 64 | 64 | 64 | 63 | 64 | 64 | 64 | 64 | 64 |
|  |  | Mn | 3.38 | 3.56 | 2.81 | 3.34 | 3.34 | 2.41 | 3.48 | 3.83 | 3.84 | 3.63 | 3.91 |
|  |  | SD | 0.75 | 0.59 | 0.85 | 0.74 | 0.84 | 1.13 | 0.69 | 0.38 | 0.44 | 0.77 | 0.34 |
| Thong Yai | Male | N | 70 | 70 | 70 | 70 | 70 | 70 | 69 | 70 | 70 | 70 | 70 |
|  |  | Mn | 3.84 | 3.86 | 3.26 | 3.44 | 3.31 | 3.16 | 3.91 | 3.86 | 3.73 | 3.80 | 3.81 |
|  |  | SD | 0.40 | 0.35 | 0.83 | 0.65 | 0.75 | 0.77 | 0.33 | 0.39 | 0.48 | 0.50 | 0.43 |
|  | Fem-ale | N | 65 | 65 | 66 | 66 | 66 | 66 | 66 | 66 | 66 | 66 | 66 |
|  |  | Mn | 3.83 | 3.82 | 3.39 | 3.68 | 3.56 | 3.30 | 3.88 | 3.91 | 3.80 | 3.77 | 3.89 |
|  |  | SD | 0.42 | 0.39 | 0.76 | 0.53 | 0.64 | 0.78 | 0.37 | 0.34 | 0.47 | 0.58 | 0.36 |
|  | Total | N | 135 | 135 | 136 | 136 | 136 | 136 | 135 | 136 | 136 | 136 | 136 |
|  |  | Mn | 3.84 | 3.84 | 3.32 | 3.56 | 3.43 | 3.23 | 3.90 | 3.88 | 3.76 | 3.79 | 3.85 |
|  |  | SD | 0.41 | 0.37 | 0.80 | 0.61 | 0.71 | 0.78 | 0.35 | 0.37 | 0.48 | 0.54 | 0.39 |
| Baan Muang 1 | Male | N | 39 | 39 | 39 | 39 | 39 | 39 | 39 | 39 | 39 | 39 | 39 |
|  |  | Mn | 3.85 | 3.85 | 3.10 | 3.54 | 3.77 | 3.26 | 3.95 | 3.87 | 3.87 | 3.77 | 3.82 |
|  |  | SD | 0.43 | 0.37 | 0.75 | 0.68 | 0.48 | 0.99 | 0.22 | 0.34 | 0.34 | 0.43 | 0.39 |
|  | Fem-ale | N | 51 | 50 | 51 | 51 | 51 | 51 | 51 | 51 | 51 | 51 | 51 |
|  |  | Mn | 3.90 | 3.90 | 3.33 | 3.61 | 3.55 | 3.16 | 3.90 | 3.86 | 3.75 | 3.75 | 3.80 |
|  |  | SD | 0.30 | 0.30 | 0.77 | 0.60 | 0.54 | 0.78 | 0.30 | 0.35 | 0.52 | 0.44 | 0.40 |
|  | Total | N | 90 | 89 | 90 | 90 | 90 | 90 | 90 | 90 | 90 | 90 | 90 |
|  |  | Mn | 3.88 | 3.88 | 3.23 | 3.58 | 3.64 | 3.20 | 3.92 | 3.87 | 3.80 | 3.76 | 3.81 |
|  |  | SD | 0.36 | 0.33 | 0.77 | 0.64 | 0.53 | 0.88 | 0.27 | 0.34 | 0.45 | 0.43 | 0.39 |
| Thong-kham Uthit 2 | Male | N | 49 | 49 | 49 | 49 | 49 | 49 | 49 | 49 | 49 | 49 | 49 |
|  |  | Mn | 3.80 | 3.80 | 2.90 | 3.08 | 3.31 | 3 | 3.86 | 3.76 | 3.82 | 3.80 | 3.92 |
|  |  | SD | 0.46 | 0.41 | 0.92 | 0.93 | 0.80 | 1 | 0.41 | 0.48 | 0.49 | 0.64 | 0.28 |
|  | Fem-ale | N | 42 | 42 | 42 | 42 | 42 | 42 | 42 | 42 | 42 | 42 | 42 |
|  |  | Mn | 3.86 | 3.81 | 2.64 | 2.98 | 3.43 | 3.10 | 3.83 | 3.74 | 3.76 | 3.81 | 3.93 |
|  |  | SD | 0.42 | 0.45 | 0.82 | 0.78 | 0.67 | 1.03 | 0.44 | 0.63 | 0.53 | 0.67 | 0.26 |
|  | Total | N | 91 | 91 | 91 | 91 | 91 | 91 | 91 | 91 | 91 | 91 | 91 |
|  |  | Mn | 3.82 | 3.80 | 2.78 | 3.03 | 3.36 | 3.04 | 3.85 | 3.75 | 3.79 | 3.80 | 3.92 |
|  |  | SD | 0.44 | 0.43 | 0.88 | 0.86 | 0.74 | 1.01 | 0.42 | 0.55 | 0.51 | 0.65 | 0.27 |
| Non Yang 2 | Male | N | 12 | 12 | 12 | 12 | 12 | 12 | 12 | 12 | 12 | 12 | 12 |
|  |  | Mn | 3.33 | 3.42 | 2.58 | 2.75 | 3.08 | 1.42 | 3.67 | 3.83 | 3.17 | 3.50 | 3.58 |
|  |  | SD | 0.65 | 0.67 | 1 | 0.87 | 0.79 | 0.90 | 0.49 | 0.39 | 0.94 | 0.52 | 0.51 |
|  | Fem-ale | N | 27 | 27 | 27 | 27 | 27 | 27 | 27 | 27 | 27 | 27 | 27 |
|  |  | Mn | 3.30 | 3.48 | 2.89 | 3 | 3.37 | 1.96 | 3.56 | 3.70 | 2.93 | 3.44 | 3.63 |
|  |  | SD | 0.72 | 0.51 | 0.93 | 0.88 | 0.79 | 0.94 | 0.51 | 0.67 | 0.83 | 0.51 | 0.49 |
|  | Total | N | 39 | 39 | 39 | 39 | 39 | 39 | 39 | 39 | 39 | 39 | 39 |
|  |  | Mn | 3.31 | 3.46 | 2.79 | 2.92 | 3.28 | 1.79 | 3.59 | 3.74 | 3 | 3.46 | 3.62 |
|  |  | SD | 0.69 | 0.55 | 0.95 | 0.87 | 0.79 | 0.95 | 0.50 | 0.59 | 0.86 | 0.51 | 0.49 |
| Nong Lek1 & 2 | Male | N | 30 | 30 | 30 | 30 | 30 | 30 | 30 | 30 | 30 | 30 | 30 |
|  |  | Mn | 3.67 | 3.67 | 3.20 | 3.60 | 3.47 | 2.90 | 3.77 | 3.77 | 3.63 | 3.57 | 3.70 |
|  |  | SD | 0.55 | 0.48 | 0.85 | 0.62 | 0.73 | 0.88 | 0.43 | 0.43 | 0.61 | 0.68 | 0.47 |
|  | Fem-ale | N | 50 | 50 | 50 | 50 | 50 | 50 | 50 | 50 | 49 | 50 | 50 |
|  |  | Mn | 3.44 | 3.66 | 3.30 | 3.60 | 3.50 | 2.76 | 3.68 | 3.82 | 3.67 | 3.50 | 3.76 |
|  |  | SD | 0.70 | 0.56 | 0.89 | 0.73 | 0.74 | 0.96 | 0.62 | 0.39 | 0.63 | 0.76 | 0.48 |
|  | Total | N | 80 | 80 | 80 | 80 | 80 | 80 | 80 | 80 | 79 | 80 | 80 |
|  |  | Mn | 3.53 | 3.66 | 3.26 | 3.60 | 3.49 | 2.81 | 3.71 | 3.80 | 3.66 | 3.53 | 3.74 |
|  |  | SD | 0.66 | 0.53 | 0.87 | 0.69 | 0.73 | 0.93 | 0.56 | 0.40 | 0.62 | 0.73 | 0.47 |
| Non Than | Male | N | 51 | 51 | 51 | 50 | 51 | 51 | 51 | 51 | 51 | 51 | 51 |
|  |  | Mn | 3.61 | 3.67 | 2.80 | 3.44 | 3.31 | 2.75 | 3.59 | 3.73 | 3.43 | 3.35 | 3.84 |
|  |  | SD | 0.67 | 0.52 | 0.85 | 0.76 | 0.81 | 0.98 | 0.67 | 0.63 | 0.70 | 0.98 | 0.37 |
|  | Fem-ale | N | 36 | 36 | 36 | 36 | 36 | 36 | 36 | 36 | 36 | 36 | 36 |
|  |  | Mn | 3.69 | 3.83 | 2.75 | 3.25 | 3.47 | 2.94 | 3.69 | 3.81 | 3.58 | 3.36 | 3.86 |
|  |  | SD | 0.58 | 0.38 | 0.77 | 0.81 | 0.70 | 1.01 | 0.67 | 0.58 | 0.60 | 0.93 | 0.42 |
|  | Total | N | 87 | 87 | 87 | 86 | 87 | 87 | 87 | 87 | 87 | 87 | 87 |
|  |  | Mn | 3.64 | 3.74 | 2.78 | 3.36 | 3.38 | 2.83 | 3.63 | 3.76 | 3.49 | 3.36 | 3.85 |
|  |  | SD | 0.63 | 0.47 | 0.81 | 0.78 | 0.77 | 0.99 | 0.67 | 0.61 | 0.66 | 0.95 | 0.39 |
| Total | Male | N | 278 | 278 | 278 | 277 | 278 | 277 | 277 | 278 | 278 | 278 | 278 |
|  |  | Mn | 3.73 | 3.76 | 3.02 | 3.38 | 3.41 | 2.91 | 3.79 | 3.81 | 3.68 | 3.65 | 3.83 |
|  |  | SD | 0.53 | 0.44 | 0.88 | 0.77 | 0.75 | 0.99 | 0.48 | 0.46 | 0.58 | 0.70 | 0.39 |
|  | Fem-ale | N | 308 | 307 | 309 | 309 | 309 | 309 | 309 | 309 | 308 | 309 | 309 |
|  |  | Mn | 3.65 | 3.73 | 3.07 | 3.40 | 3.46 | 2.89 | 3.74 | 3.82 | 3.67 | 3.64 | 3.83 |
|  |  | SD | 0.62 | 0.49 | 0.86 | 0.75 | 0.70 | 1.03 | 0.54 | 0.47 | 0.60 | 0.67 | 0.41 |
|  | Total | N | 586 | 585 | 587 | 586 | 587 | 586 | 586 | 587 | 586 | 587 | 587 |
|  |  | Mn | 3.68 | 3.74 | 3.05 | 3.39 | 3.43 | 2.90 | 3.76 | 3.81 | 3.68 | 3.65 | 3.83 |
|  |  | SD | 0.58 | 0.47 | 0.87 | 0.76 | 0.73 | 1.01 | 0.51 | 0.46 | 0.59 | 0.69 | 0.40 |

# Subjective wellbeing descriptives

## Table 2: Subjective wellbeing scores by neighbourhood broken down by gender.

|  |  | All | | Men | | Women | |
| --- | --- | --- | --- | --- | --- | --- | --- |
| Location Code | | SWEMWB SCORES | PS SCORES | SWEMWB SCORES | PS SCORES | SWEMWB SCORES | PS SCORES |
| CBD | Mn | 27.32 | 17.93 | 27.97 | 16.65 | 26.35 | 19.83 |
|  | N | 57 | 57 | 34 | 34 | 23 | 23 |
|  | SD | 5.26 | 5.91 | 4.26 | 5.31 | 6.46 | 6.34 |
| Free Area | Mn | 27.97 | 18.09 | 27.76 | 18.03 | 28.18 | 18.15 |
|  | N | 78 | 78 | 38 | 38 | 40 | 40 |
|  | SD | 4.41 | 4.41 | 4.51 | 4.42 | 4.36 | 4.46 |
| Kaptembwo | Mn | 27.26 | 19.53 | 28.09 | 18.73 | 26.41 | 20.36 |
|  | N | 130 | 130 | 66 | 66 | 64 | 64 |
|  | SD | 5.10 | 4.66 | 4.69 | 4.20 | 5.39 | 4.98 |
| London | Mn | 28.64 | 17.75 | 28.85 | 17.67 | 28.46 | 17.82 |
|  | N | 113 | 113 | 52 | 52 | 61 | 61 |
|  | SD | 4.13 | 4.62 | 4.02 | 5.15 | 4.24 | 4.15 |
| Section No58 | Mn | 28.24 | 17.62 | 28.33 | 17.69 | 28.13 | 17.53 |
|  | N | 100 | 100 | 55 | 55 | 45 | 45 |
|  | SD | 4.63 | 4.36 | 4.13 | 4.48 | 5.22 | 4.27 |
| Shabab | Mn | 27.30 | 18.24 | 26.32 | 19.80 | 28.28 | 16.68 |
|  | N | 50 | 50 | 25 | 25 | 25 | 25 |
|  | SD | 5.14 | 6.14 | 5.79 | 4.98 | 4.29 | 6.87 |
| Total | Mn | 27.86 | 18.28 | 28.06 | 18.05 | 27.64 | 18.52 |
|  | N | 528 | 528 | 270 | 270 | 258 | 258 |
|  | SD | 4.75 | 4.90 | 4.50 | 4.73 | 5 | 5.07 |

|  |  | All | | Men | | Women | |
| --- | --- | --- | --- | --- | --- | --- | --- |
| Location Code | | SWEMWB SCORES | PS SCORES | SWEMWB SCORES | PS SCORES | SWEMWB SCORES | PS SCORES |
| Baan Non | Mn | 28.44 | 14.83 | 27.56 | 15.67 | 29.08 | 14.22 |
|  | N | 64 | 64 | 27 | 27 | 37 | 37 |
|  | SD | 4.08 | 4.44 | 4.09 | 5.06 | 4.01 | 3.89 |
| Thong Yai | Mn | 27.68 | 14.57 | 27.84 | 14.81 | 27.52 | 14.30 |
|  | N | 136 | 136 | 70 | 70 | 66 | 66 |
|  | SD | 4.53 | 4.74 | 4.54 | 4.52 | 4.55 | 4.99 |
| Baan Muang 1 | Mn | 27.99 | 14.01 | 26.41 | 15.49 | 29.20 | 12.88 |
|  | N | 90 | 90 | 39 | 39 | 51 | 51 |
|  | SD | 4.24 | 4.98 | 4.60 | 5.03 | 3.53 | 4.68 |
| Thongkham Uthit 2 | Mn | 28.55 | 13.31 | 28.86 | 12.61 | 28.19 | 14.12 |
|  | N | 91 | 91 | 49 | 49 | 42 | 42 |
|  | SD | 4.69 | 5.39 | 5.23 | 6.14 | 4 | 4.28 |
| Non Yang 2 | Mn | 27.62 | 14.03 | 28.25 | 14.67 | 27.33 | 13.74 |
|  | N | 39 | 39 | 12 | 12 | 27 | 27 |
|  | SD | 3.86 | 4.78 | 3.89 | 4.79 | 3.89 | 4.83 |
| Nong Lek1 & 2 | Mn | 28.91 | 14.08 | 29.63 | 14.10 | 28.48 | 14.06 |
|  | N | 80 | 80 | 30 | 30 | 50 | 50 |
|  | SD | 3.94 | 4.52 | 2.98 | 3.90 | 4.39 | 4.90 |
| Non Than | Mn | 27.84 | 14.55 | 27.27 | 14.43 | 28.64 | 14.72 |
|  | N | 87 | 87 | 51 | 51 | 36 | 36 |
|  | SD | 4 | 4.59 | 3.90 | 5.02 | 4.06 | 3.97 |
| Total | Mn | 28.13 | 14.21 | 27.90 | 14.45 | 28.34 | 13.99 |
|  | N | 587 | 587 | 278 | 278 | 309 | 309 |
|  | SD | 4.27 | 4.80 | 4.41 | 5.05 | 4.13 | 4.57 |

# Transect walk results comparison

### Starting wellbeing assessments

Two survey tools were used to assess underlying mood (SWEMWB) and stress levels (PS).

### SWEMWB


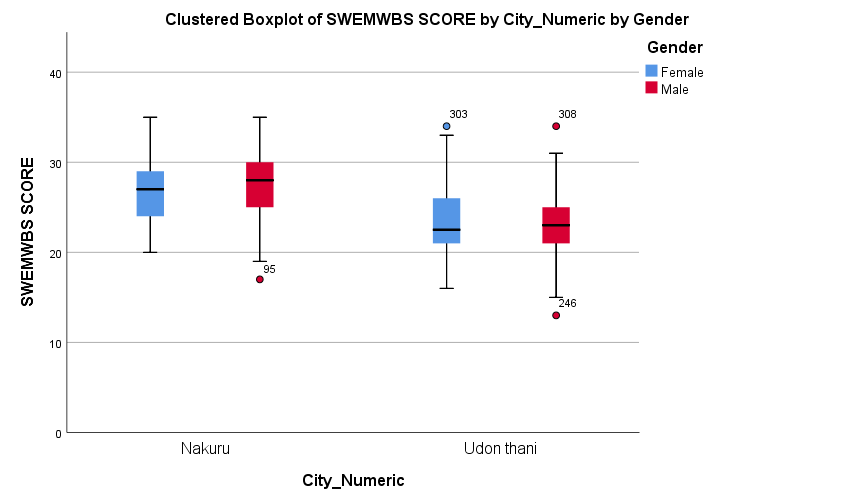


Results indicate a statistically significant overall higher subjective wellbeing for residents in Nakuru compared to Udon thani (t(232)=7.386, p=0)). Investigating this further by gender indicates that both women and men have higher overall wellbeing in Nakuru than Udon thani (Women (t(113)=4.177, p=0); Men (t(117)=6.214, p=0)).

### PS


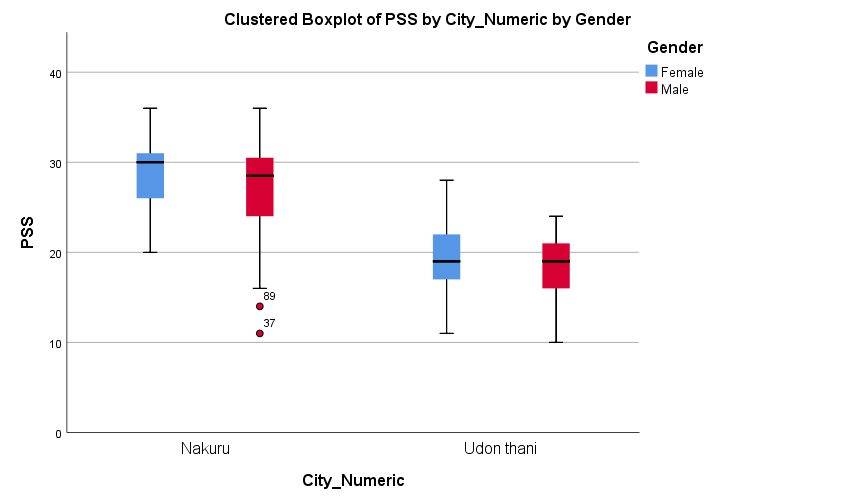


Conversely the PS indicate that stress levels in Udon thani are significantly lower than in Nakuru (for both genders) (t(231)=16.233, p=00)). Investigating this by gender both women (t(114)=14.025, p=00) and men (t(115)=9.877, p=00) have statistically lower stress in Udon than Nakuru.

# Udon Thani Transect Walk Findings

## Subjective Scales

### UWIST MACL

Transect walk participants experienced a significant effect on their hedonic tone (t(114)=2.707, p=08) with a lower mood at the end of the walk than at the outset independent of route (pre-Mn=23.38; post-Mn=22.57). There was a statistically weaker effect on arousal (t(114)=1.924, p=0.057) with lower levels of physiological alertness at the end of the walk (pre-Mn=21.48; post-Mn=20.82).

Looking at the influence of route on the UWIST scores, participants who began their transect walk in the park and ended in the market did not see a significant change in hedonic tone (t(49)=0.081, p=0.936). However, participants who began their transect walk in the market and ended in the park saw a significant decrease (pre-Mn=23.74; post-Mn=22.32) in hedonic tone (t(64)=3.908, p=00).

Investigating this with a gender breakdown reveals a significant drop in hedonic tone for both women (pre-Mn=23.98; post-Mn=22.71) (t(44)=2.735, p=09) and men (pre-Mn=23.20; post-Mn=21.45) (t(19)=3.144, p=05) starting in the market and ending in the park. There are no significant effects for those undertaken the inverse route (Park to Market). These results contradict most of the evidence from Europe which have found positive effects from green space on mood.

###
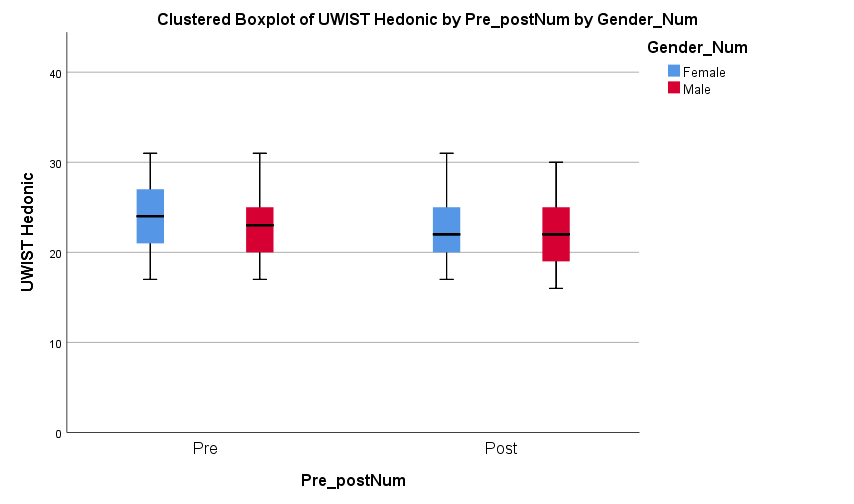
Udon Thani transect walk route

####
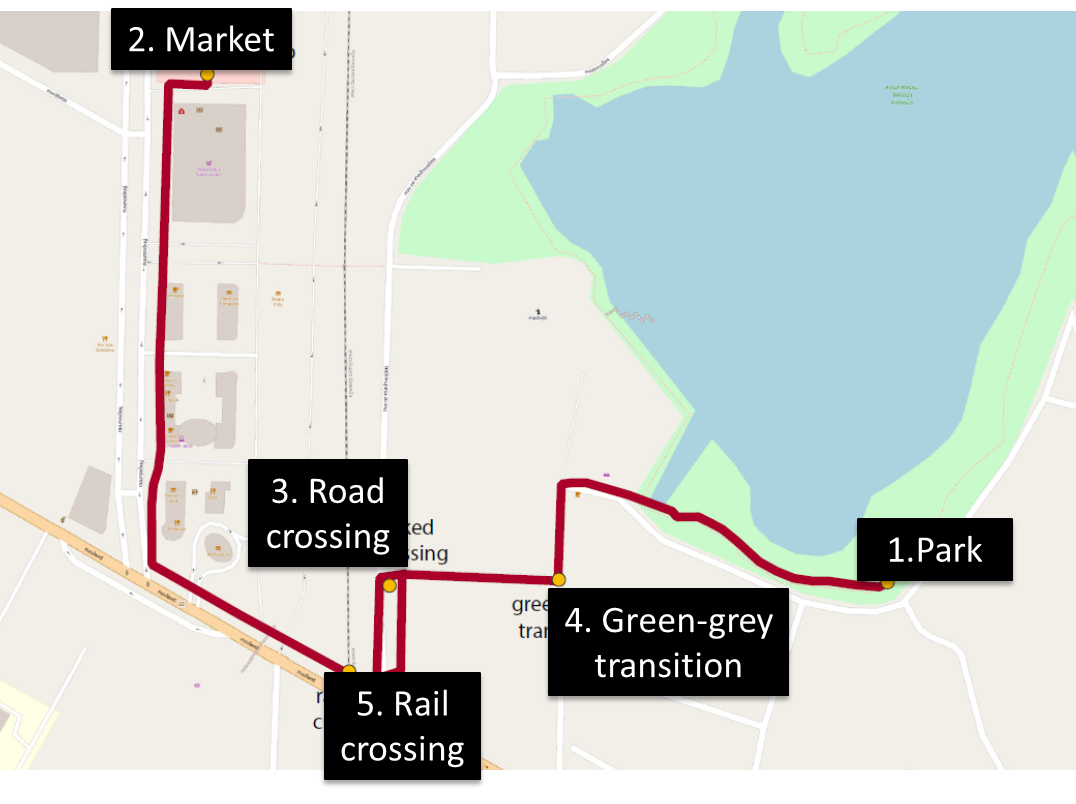


# Nakuru Transect Walk Findings

## Subjective Scales

### UWIST MACL

In Nakuru the only statistically significant effect is on men who experience a significant increase in hedonic tone (pre-Mn=21.41; post-Mn=22.38) when ending their walk in the park (t(31)=-2.142, p=0.040).


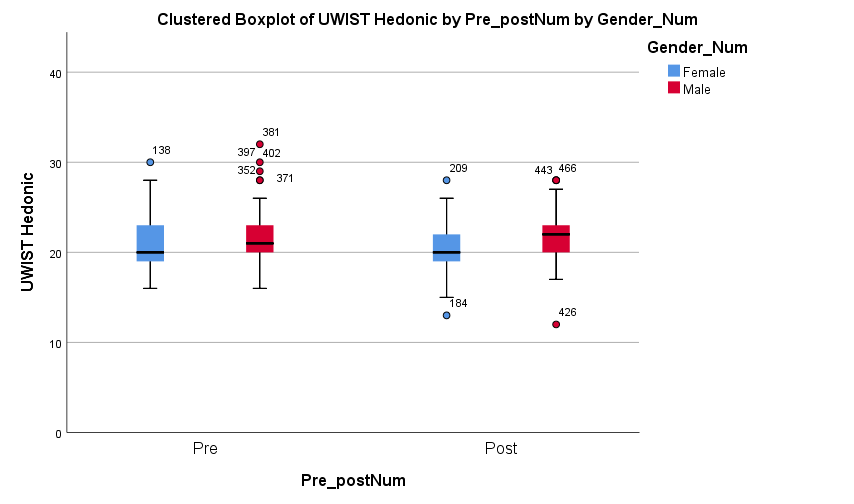


#### Nakuru transect walk route


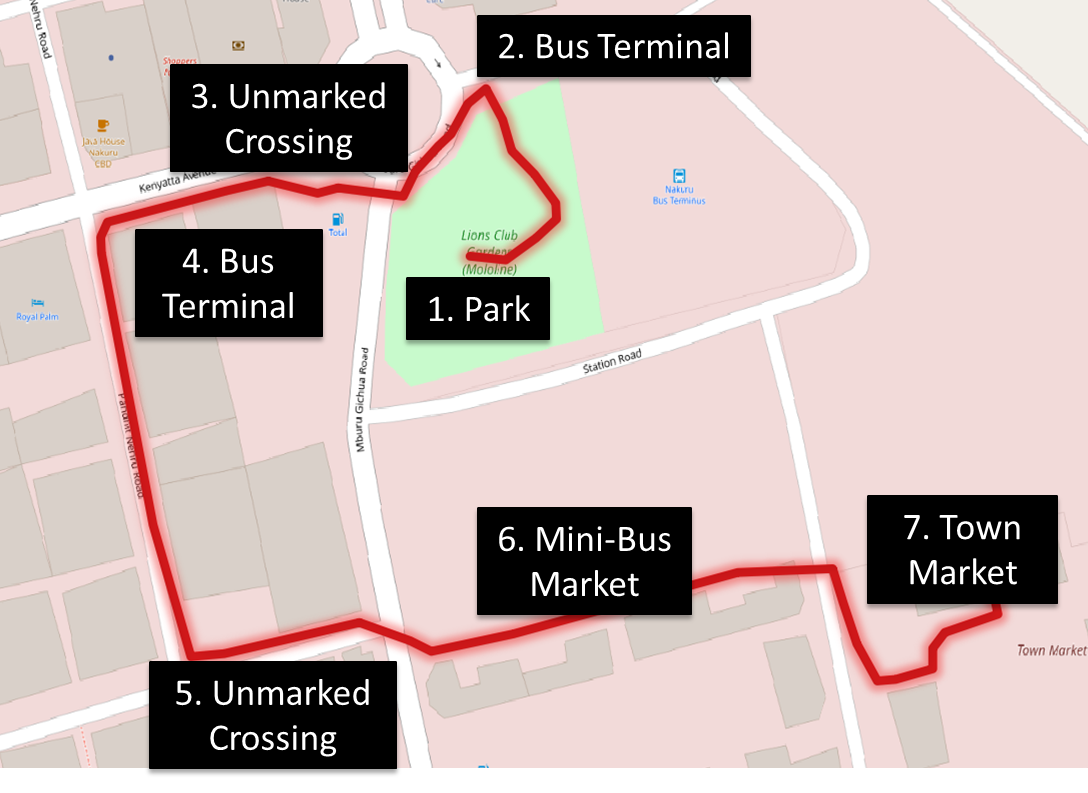


1. Corburn, J. Urban place and health equity: Critical issues and practices. *Int. J. Environ. Res. Public Health* **14**, 1–10 (2017).

2. Ryan, R. M. & Deci, E. L. On Happiness and Human Potentials: A Review of Research on Hedonic and Eudaimonic Well-Being. *Annu. Rev. Psychol.* **52**, 141–166 (2001).

3. Huber, M. *et al.* How should we define health? *BMJ* **343**, d4163 (2011).

4. Haver, A., Akerjordet, K., Caputi, P., Furunes, T. & Magee, C. Measuring mental well-being: A validation of the Short Warwick–Edinburgh Mental Well-Being Scale in Norwegian and Swedish. *Scand. J. Public Health* **43**, 721–727 (2015).

5. Tennant, R. *et al.* The Warwick-Edinburgh Mental Well-being Scale (WEMWBS): development and UK validation. *Health Qual. Life Outcomes* **5**, 63 (2007).

6. Stewart-Brown, S. *et al.* Internal construct validity of the Warwick-Edinburgh Mental Well-Being Scale (WEMWBS): A Rasch analysis using data from the Scottish Health Education Population Survey. *Health Qual. Life Outcomes* **7**, 1–8 (2009).

7. Ward Thompson, C. *et al.* More green space is linked to less stress in deprived communities: Evidence from salivary cortisol patterns. *Landsc. Urban Plan.* **105**, 221–229 (2012).

8. Cohen, S., Kamarack, T. & Mermelstein, R. *Perceived Stress Scale , PS: Quick Facts about the Tool*. (2014).

9. Cohen, S., Kamarck, T. & Mermelstein, R. A Global Measure of Perceived Stress. *J. Health Soc. Behav.* **24**, 385–396 (2014).

10. Shaffer, F. & Ginsberg, J. P. An Overview of Heart Rate Variability Metrics and Norms. *Front. Public Heal.* **5**, 1–17 (2017).
